# PRMT3 drives PD-L1-mediated immune escape through activating PDHK1-regulated glycolysis in hepatocellular carcinoma

**DOI:** 10.1038/s41419-025-07482-7

**Published:** 2025-03-06

**Authors:** Chen-Hong Ding, Fang-Zhi Yan, Bo-Nan Xu, Hui Qian, Xia-Lu Hong, Shu-Qing Liu, Yuan-Yuan Luo, Si-Han Wu, Ling-Yan Cai, Xin Zhang, Wei-Fen Xie

**Affiliations:** 1 Department of Gastroenterology, Shanghai East Hospital, Tongji University School of Medicine, Shanghai, China; 2 Department of Gastroenterology, Changzheng Hospital, Naval Medical University, Shanghai, China

**Keywords:** Cancer metabolism, Tumour immunology

## Abstract

Aberrant expression of programmed death ligand-1 (PD-L1) facilitates tumor immune evasion. Protein arginine methyltransferase 3 (PRMT3), a member of type I PRMT family, mediates asymmetric dimethylarginine (ADMA) modification of various substrate proteins. This study investigates the role of PRMT3 in PD-L1-associated tumor immunosuppression in hepatocellular carcinoma (HCC). Hepatocyte-specific knockout of *Prmt3* significantly suppressed HCC progression in DEN-CCL_4_-treated mice. Knockout of *Prmt3* in HCC cells markedly increased CD8^+^ T cell infiltration, and reduced lactate production in tumors. PRMT3 interacted with pyruvate dehydrogenase kinase 1 (PDHK1), asymmetric dimethylation of PDHK1 at arginine 363 and 368 residues and increased its kinase activity. The R363/368 K mutant or inhibition of PDHK1 by JX06 blocked the effect of PRMT3 on lactate production. JX06 treatment also attenuated the tumor-promoting role of PRMT3 in HCC in vitro and in vivo. Furthermore, RNA-seq analysis revealed that knockout of PRMT3 downregulates the tumor-associated immune checkpoint, PD-L1, in tumor tissues. Chromatin immunoprecipitation (ChIP) assay demonstrated that PRMT3 promotes lactate-induced PD-L1 expression by enhancing the direct binding of histone H3 lysine 18 lactylation (H3K18la) to the PD-L1 promoter. Tissue microarray analysis showed a positive correlation between PRMT3 and PD-L1 expression in HCC patients. Anti-PD-L1 treatment reversed PRMT3-induced tumor growth and restored CD8^+^ T cell infiltration. Our research links PRMT3-mediated metabolic reprogramming and immune evasion, revealing that the PRMT3-PDHK1-lactate-PD-L1 axis may be a potential target for improving the efficacy of immunotherapy in HCC.

## Introduction

Hepatocellular carcinoma (HCC), the primary form of liver cancer, is the third leading cause of cancer-related mortality worldwide [1]. For the advanced HCC patients who are not suitable for surgery, chemotherapy, radiotherapy, targeted therapy, and immunotherapy are the main treatment options. Immune checkpoint blockade (ICB), particularly targeting programmed cell death protein 1 (PD-1) and its ligand PD-L1, has demonstrated promising clinical benefits as monotherapy for advanced HCC patients, and combining it with other therapeutic agents may further enhance its effectiveness [2–4]. The IMbrave150 trial revealed that atezolizumab plus bevacizumab offered unprecedented overall survival (OS) benefits compared to sorafenib [2]. However, other ICB therapy clinical trials, had limited benefit, highlighting the need to further investigate mechanisms of immunotherapy [3, 4]. PD-L1, which is often expressed in tumor cells, interacts with PD-1 on infiltrating immune cells, thereby facilitating tumor immune evasion [5]. Extensive research aimed at identifying more effective immune checkpoint molecules has shown that tumor PD-L1 expression is still a promising but not perfect biomarker for predicting immunotherapy response [6, 7]. Therefore, further investigation into the molecular mechanisms regulating PD-L1 expression is essential for developing foundational strategies in ICB therapy and improving the effectiveness of immunotherapy for HCC patients.

Metabolic dysfunction plays a critical role in tumor development and affects the efficacy of immunotherapy [8]. A hallmark of cancer, aerobic glycolysis—commonly known as the Warburg effect—characterizes the energy metabolism of most tumor cells [9]. This metabolic shift allows tumor cells to rapidly proliferate by utilizing glucose, even in the presence of oxygen, and results in the production and release of large amounts of lactate into the tumor microenvironment. Accumulated lactate significantly impacts tumor progression, correlating with increased metastasis and poorer survival outcomes [10]. Previous studies have shown that lactate creates an immune-tolerant microenvironment by impairing various immune cells. Lactate suppresses the cytotoxic function of T cells and reduces the secretion of Interferon gamma (IFN-γ) by Natural Killer cells [11]. Recent studies also revealed that glycolytic-dominant metabolic reprogramming influences PD-L1 expression. For example, tumor-derived exosomes induced glucose metabolic reprogramming in macrophages drives PD-L1 expression through NF-kB activation [12]. In lung cancer cells, lactate promotes PD-L1 expression by activating G protein-coupled receptor 81 [13]. Notably, lactylation, a lactate-mediated modification of proteins, particularly histone lactylation, has emerged as a novel epigenetic modification that promotes downstream gene transcription and contributes to tumor development [14–16]. However, the role of histone lactylation, which connects metabolic and epigenetic regulation, in HCC tumor immunity remains not fully understood.

The family of protein arginine methyltransferases (PRMTs) plays a crucial role in various cellular processes and diseases by mediating methylation modifications on arginine residues of substrate proteins [17–21]. In our previous study investigating the involvement of PRMTs in HCC, we observed a notable upregulation of PRMT3 expression in HCC tissues, suggesting that PRMT3 may play a functional role in HCC [22]. PRMT3, a type I PRMT, primarily catalyzes the formation of asymmetric dimethylarginine (ADMA) on both histone and non-histone proteins. Elevated PRMT3 expression has been observed in various tumors and is linked to poor clinical outcomes, including in pancreatic cancer, glioblastoma, and HCC [23–26]. Previous studies have identified numerous metabolic enzymes interacting with PRMT3, indicating PRMT3’s involvement in metabolic modulation [27]. A recent report showed that PRMT3-mediated methylation of glyceraldehyde-3-phosphate dehydrogenase (GAPDH) at R248 enhances its catalytic activity, thus increasing glycolysis in pancreatic cancer cells [23]. Another study demonstrates that PRMT3 promotes aerobic glycolysis by methylating lactate dehydrogenase A (LDHA) at R112 in HCC cells [26]. Despite these findings, the role of PRMT3 in HCC and its impact on tumor immunity through metabolic reprogramming remain underexplored.

In this study, we found that knockout of *Prmt3* significantly suppressed HCC progression in mouse HCC models, accompanied by a marked increase in CD8^+^ T cell infiltration and a reduction in lactate production within tumors. We identified pyruvate dehydrogenase kinase 1 (PDHK1) as a substrate of PRMT3 that is associated aerobic glycolysis. PRMT3 promotes HCC growth through asymmetric dimethylation of PDHK1 at arginine 363/368, resulting in enhanced lactate accumulation. This elevated lactate subsequently facilitates histone lactylation, which epigenetically upregulates PD-L1 expression by increasing the binding of histone H3 lysine 18 lactylation (H3K18la) at the PD-L1 promoter. Importantly, treatment with anti-PD-L1 antibodies inhibited the oncogenic effects of PRMT3 and restored CD8^+^ T cell infiltration. Our research establishes a mechanistic link between PRMT3-mediated metabolic reprogramming and tumor immunity.

## Results

### PRMT3 plays oncogenic role in HCC

Previous studies have shown that PRMT3 is upregulated in human HCC tissues and promotes the proliferation of HCC cells [26]. Consistent with these findings, we observed a significant increase in PRMT3 expression in HCC tissues. High PRMT3 expression was correlated with poor prognosis in HCC patients (Supplementary Fig. S1A–1C). In Huh7 cells, overexpression of PRMT3 promotes proliferation, migration, and invasion, while knockdown of PRMT3 or targeting PRMT3 with SGC707 effectively suppresses these malignant phenotypes (Supplementary Fig. S1D–1F). Collectively, these results demonstrated the oncogenic role of PRMT3 in HCC.

### Knockout of *Prmt3* suppresses tumor progression and increases CD8^+^ T cell infiltration in mouse tumors

**Fig. 1:**
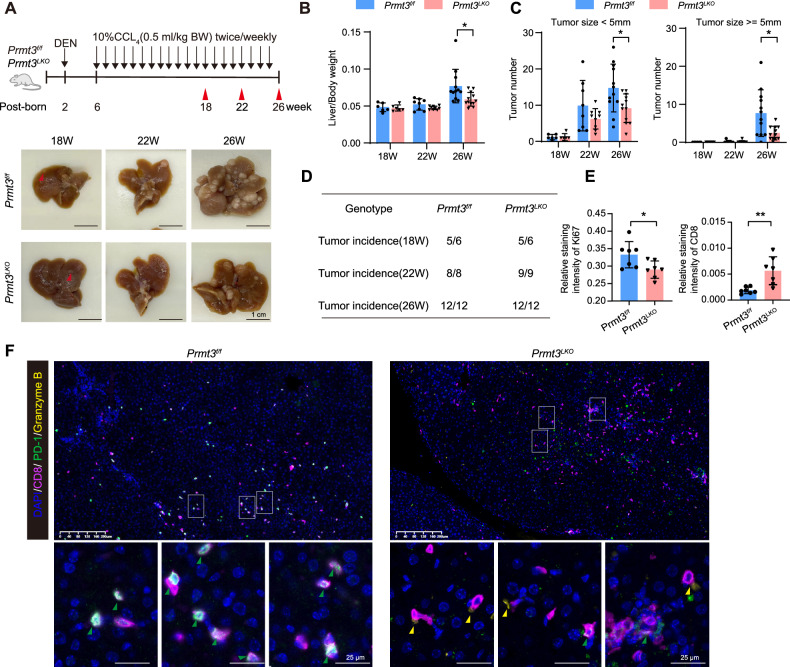
*Prmt3* deletion suppresses tumor progression and increases CD8^+^ T cell infiltration in mouse tumors. **A** Schematic depiction of the HCC model induced by DEN-CCl_4_ (upper). Representative images of livers derived from *Prmt3*^*f/f*^ and *Prmt3*^*LKO*^ mice at different time points (lower). Red arrows indicate tumor nodes. Scale bar, 1 cm. **B** Liver/body weight ratio in *Prmt3*^*f/f*^ and *Prmt3*^*LKO*^ mice at different time points. *P < 0.05. **C** Number and size of tumor nodules on the liver surface in both groups. *P < 0.05. **D** Tumor incidence scored at different time points in both groups. The number of mice recorded at different time points in **B**, **C**, and **D** was consistent with the number shown in **D**. **E** Statistical analysis of Ki67 and CD8 expression in tumor tissues of *Prmt3*^*f/f*^ and *Prmt3*^*LKO*^ mice at 26 weeks via immunohistochemistry. *n* = 7 in each group. * *P* < 0.05, ** *P* < 0.01. **F** Representative images of CD8, PD-1, and Granzyme B (GrB) detection via multiplex immunofluorescences in tumors of *Prmt3*^*f/f*^ and *Prmt3*^*LKO*^ mice. Green arrows indicate PD-1^+^ CD8^+^ cells and yellow arrows indicate GrB^+^CD8^+^ cells. Scale bar, 25 μm. The data are presented as mean ± SD, and the statistical tests were all two-tailed.

The liver-specific Prmt3 knockout mouse (termed as *Prmt3*^*LKO*^) were generated by mating the albumin-Cre (Alb-Cre) mice with *Prmt3*^*f/f*^ mice. To evaluate the role of PRMT3 in hepatocarcinogenesis, primary HCC was induced by treatment with diethylnitrosamine (DEN) and carbon tetrachloride (CCl_4_) in *Prmt3*^*f/f*^ and *Prmt3*^*LKO*^ mice (Fig. 1A, Supplementary Fig. S2A). Tumor nodules appeared in the livers of both *Prmt3*^*f/f*^ and *Prmt3*^*LKO*^ mice at 18-week-old. The liver-to-body weight ratio remained unchanged in both groups of mice. With extended DEN-CCl_4_ treatment, deletion of *Prmt3* significantly reduced tumor burden, as evidenced by liver/body weight ratio, tumor number, and tumor size, but did not affect the incidence of tumor formation at 26-week-old (Fig. 1B–D). Ki67 staining further confirmed that tumor cell proliferation decreased in 26-week-old *Prmt3*^*LKO*^ mice. Notably, CD8^+^ T cell infiltration in the tumors of *Prmt3*^*LKO*^ mice was markedly increased compared to *Prmt3*^*f/f*^ mice (Fig. 1E, Supplementary Fig. S2B). Additionally, multiplex immunofluorescence results indicated that *Prmt3* knockout enhanced the proportion of activated CD8^+^ T cells (Granzyme B^+^CD8^+^) while decreased the proportion of exhausted CD8^+^ T cells (PD-1^+^ CD8^+^) infiltrating tumors (Fig. 1F). To evaluate the role of T cells in the impact of Prmt3 on tumor growth, *Prmt3* knockout (*Prmt3*^*KO*^) and control Hepa1-6 cells were inoculated into BALB/c-nu/nu (nude) mice and immunocompetent C57BL/6 mice. Consistently, the growth of *Prmt3*^*KO*^ HCC xenograft was significantly reduced, nearly eliminated, in C57BL/6 mice compared to that in nude mice (Supplementary Fig. S2C–E). Additionally, *Prmt3* knockout also enhanced CD8^+^ T cell infiltration in Hepa1-6 tumors derived from C57BL/6 mice (Supplementary Fig. S2F). These results suggest that T cell infiltration contributes to the inhibitory effect of *Prmt3* knockout on tumor growth.

### PRMT3 promotes lactate accumulation through methylation of PDHK1

**Fig. 2:**
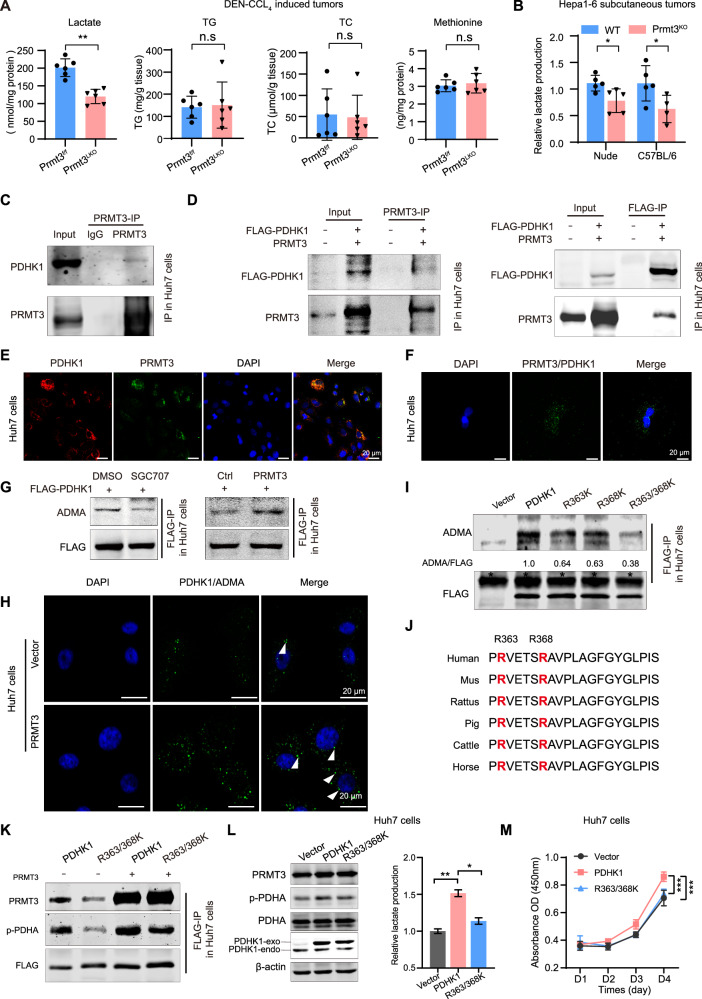
PRMT3 promotes lactate accumulation by methylating PDHK1. **A** Lactate levels, triglyceride (TG) levels, total cholesterol (TC) levels, and methionine levels were detected in tumors derived from DEN-CCL_4_-induced HCC. *n* = 6 in each group. ** *P* < 0.01, n.s, no significant difference. **B** Lactate levels were detected in subcutaneous Hepa1-6 tumors derived from nude mice and C57BL/6 mice. *n* = 5 or 4 in each group. Each point represents data from one individual mouse. * *P* < 0.05. **C** Cell extracts from Huh7 cells with PRMT3 overexpression were subjected to immunoprecipitation using an anti-PRMT3 antibody, followed by immunoblotting to detect PDHK1. **D** The binding of PRMT3 and PDHK1 in Huh7 cells transfected with HA-PRMT3 and FLAG-PDHK1 were verified by reciprocal immunoprecipitation assays. **E** The colocalization of PRMT3 and PDHK1 in Huh7 cells was measured by immunofluorescence assay. Scale bar, 20 μm. Pearson’s correlation coefficient (PCC) for evaluating the colocalization of PRMT3 and PDHK1 was analyzed using ImageJ software [72]. The mean PCC R value obtained was 0.82 (R = 0.82). **F** Representative images of PLA performed in Huh7 cells using antibodies against PDHK1 and PRMT3 were shown. Scale bar, 20 μm. The green dots indicated PDHK1 interacting with PRMT3. **G** PDHK1 was immunoprecipitated from Huh7 cells treated as indicated, and ADMA levels were subsequently detected by immunoblot analysis. **H** Representative images of PLA performed in Huh7 cells with PRMT3 overexpression using antibodies against PDHK1 and ADMA were shown. Scale bar, 20 μm. The green dots, as indicated by the white arrows, represent PDHK1 that has undergone ADMA modification. **I** Wildtype PDHK1, R363K mutant, R368K mutant, R363/ R368K mutant, and their corresponding vector control were transfected into Huh7 cells with PRMT3 overexpression. Subsequently, these PDHK1 variants were immunoprecipitated using Anti-FLAG beads and subjected to immunoblot analysis to detect the level of ADMA. * indicated heavy chain. **J** The amino acid sequences containing the R363 and R368 sites in various species. **K** Wildtype PDHK1 and R363/R368K were transfected in Huh7 cells with or without PRMT3 overexpression for 48 hours. Subsequently, PDHK1 and R363/R368K mutations were immunoprecipitated with Anti-FLAG beads and subjected to immunoblot analysis to detect the p-PDHA level. **L** Wildtype PDHK1, R363/R368K, and their corresponding vector control were transfected in Huh7 cells with PRMT3 overexpression for 48 hours. Then the levels of PDHA, p-PDHA and lactate were detected. **M** Wildtype PDHK1, R363/R368K, and their corresponding vector control were transfected in Huh7 cells with PRMT3 overexpression for 12 hours. Subsequently, the cells were seeded into a 96-well plate at a density of 5000 cells per well to assess proliferation at the indicated time points. The data are presented as mean ± SD, and the statistical tests were all two-tailed.

Previous research has established a strong link between tumor immunity and metabolic alterations, including glucose, fatty acid, cholesterol, and amino acid metabolism [8, 28–30]. Methionine metabolism generates S-Adenosylmethionine (SAM), which serve as the methyl donor facilitating methylation modifications carried out by PRMTs [31]. To identify metabolites linked to CD8^+^ T cell infiltration, we then analyzed lactate, triglycerides (TG), total cholesterol (TC), and methionine levels in DEN-CCl_4_-induced tumors from *Prmt3*^*f/f*^ mice and *Prmt3*^*LK*O^ mice. We found that lactate production was significantly reduced in *Prmt3*^*LK*O^ mice compared to *Prmt3*^*f/f*^ mice. Conversely, *Prmt3* depletion did not significantly affect TG, TC, or methionine levels (Fig. 2A). Additionally, in Hepa1-6 subcutaneous tumors derived from either nude mice or C57BL/6 mice, *Prmt3* knockout also significantly decreased lactate production (Fig. 2B). These results indicate that knockout of *Prmt3* reduced lactate production, which may contribute to the increased CD8^+^ T cell infiltration in tumors.

Next, we investigated the molecular mechanism of PRMT3 in driving lactate accumulation. In Hepa1-6 cells, knockout of Prmt3 inhibited cellular malignancy phenotypes, including proliferation, migration, and invasion and was associated with decreased intracellular lactate accumulation, while elevated Prmt3 expression exerted the opposite effect (Supplementary Fig. S3A–C). In Huh7 cells, lactate accumulation and the activation of glycolysis were observed upon PRMT3 overexpression suggested that PRMT3 is involved in regulating the glycolytic metabolism (Supplementary Fig. S3D–G). It is well-documented that aberrantly activated PDHK1 phosphorylates and inactivates the pyruvate dehydrogenase complex (PDC), which impedes the oxidative phosphorylation pathway and promotes glycolysis in tumors (Supplementary Fig. S3H) [32]. Reciprocal immunoprecipitation assays (IP) revealed the interaction between PRMT3 and PDHK1, a key regulator of glycolysis in Huh7 cells (Fig. 2C–D). Immunofluorescence (IF) and proximity ligation assays (PLA) further confirmed the binding of endogenous PRMT3 to PDHK1 (Fig. 2E–F). This interaction suggested that PDHK1 is the potential substrate of PRMT3. We further evaluated the PRMT3-catalyzed ADMA modification of PDHK1 using immunoprecipitation followed by immunoblotting assays (IP-IB). IP-IB analysis showed that inhibiting PRMT3 reduced, whereas upregulating PRMT3 increased the ADMA modification of PDHK1 in Huh7 cells (Fig. 2G). Moreover, PLA assay using PDHK1 and ADMA antibodies also confirmed that PRMT3 promotes the ADMA modification of PDHK1(Fig. 2H).

To identify methylation sites, we conducted mass spectrometry (MS) analysis on PDHK1 proteins immunoprecipitated from Huh7 cells with PRMT3 overexpression. However, PDHK1 protein (NP_001265478.1), rich in arginine (R) and lysine (K) residues (over 50 within its 434 amino acid sequence), tends to fragment into small peptides ( < 6 aa) that are challenging to identify via MS. To address this issue, we generated a series of PDHK1 mutations (R2/5/8 K, R86K, R164/166/168 K, R208/212 K, R233K, R257/258 K, R315K, R339K, R363/368 K) substituting all R residues with K, excluding MS-identified peptides (Supplementary Table S1). IP-IB results revealed that R363/368 K mutation significantly reduced the ADMA level of PDHK1 (Supplementary Fig. S3I, Fig. 2I). The high conservation of arginine residue sequence across species suggests a potential biological function for this methylation (Fig. 2J). Previous studies have demonstrated that activated PDHK1 enhances its binding to its substrate PDH, increased phosphorylation of PDH at the serine 293 site (termed as p-PDHA in this paper) [33]. Our IP results showed that PRMT3 significantly increased PDHK1-mediated phosphorylated PDHA, whereas the R363/368 K mutant attenuated this effect (Fig. 2K). Moreover, the R363/368 K mutant decreased the level of p-PDHA, reduced lactate production, and inhibited tumor cell proliferation in Huh7 cells compared to wild-type PDHK1 (Fig. 2L-M). Collectively, these results indicate that PRMT3 promotes lactate accumulation by methylating PDHK1.

### Methylation of PDHK1 is responsible for the tumor-promoting effects of PRMT3 in HCC

**Fig. 3:**
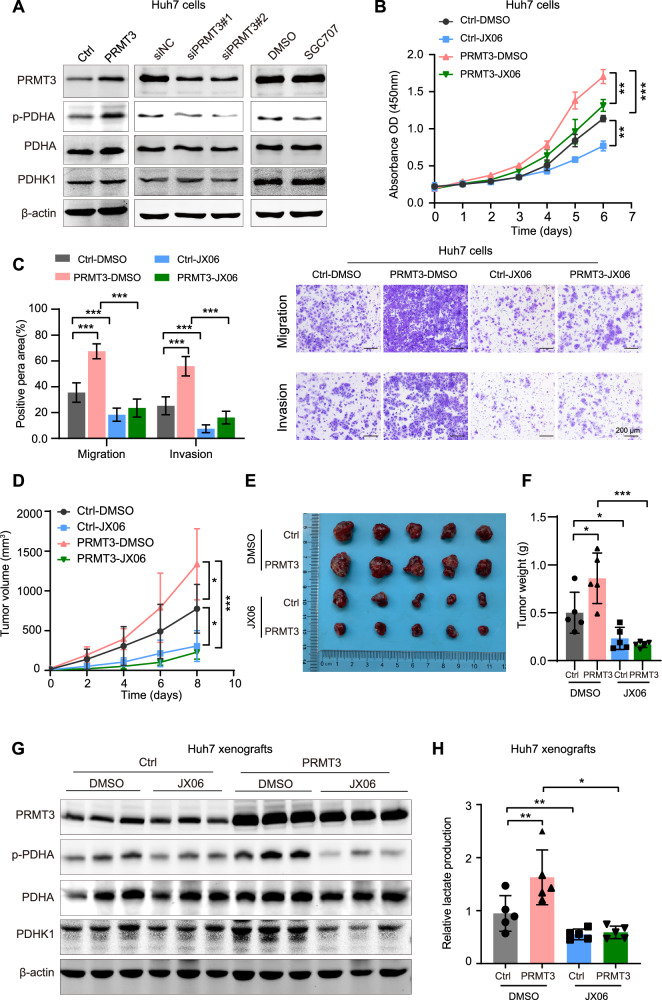
Methylation of PDHK1 is responsible for the oncogenic role of PRMT3 in HCC. **A** Immunoblot analysis of the PDHA and phosphorylated PDHA levels in Huh7 cells with indicated treatments. The proliferation capacity (**B**), and migration and invasion capacity (**C**) of the indicated cells were evaluated. Huh7 cells were infected with Lenti-PRMT3 or Lenti-Ctrl and then treated with JX06 (200 nM) or DMSO. The representative images of stained transwell chamber were shown at the right of Fig. 3C. Scale bar, 200 μm. ** *P* < 0.01, *** *P* < 0.001. The tumor growth curve (**D**), tumor images (**E**), and tumor weight (**F**) of Huh7 xenografts were shown. *n* = 5 in each group. Huh7 cells infected with Lenti-PRMT3 or Lenti-Ctrl were inoculated into nude mice. Tumor-bearing mice were then administered JX06 or vehicle via intraperitoneal injection every 2 days at a dose of 30 mg/kg. * *P* < 0.05, *** *P* < 0.001. **G** Immunoblot analysis of PRMT3, PDHK1, and p-PDHA (phosphorylated PDHA) levels in Huh7 xenografts derived from nude mice. **H** Lactate production was measured in Huh7 xenografts obtained from nude mice. *n* = 5 in each group. * *P* < 0.05, ** *P* < 0.01. The data are presented as mean ± SD, and the statistical tests were all two-tailed.

To assess the impact of PDHK1’s methylation activity involved in the oncogenic role of PRMT3 in HCC, we analyzed the levels of p-PDHA in Huh7 cells. Immunoblot analysis revealed that upregulation of PRMT3 increases p-PDHA, while inhibition or downregulation of PRMT3 significantly reduces p-PDHA without affecting PDHK1 protein levels (Fig. 3A). SGC707 treatment also reduced p-PDHA levels and lactate levels in Huh7 xenografts (Supplementary Fig. S4A-B). These findings suggest PRMT3 increases the endogenous PDHK1 activity. We then employed the PDHK1 inhibitor JX06 to investigate the role of PDHK1 in PRMT3 function [34]. In Huh7 cells, JX06 treatment effectively abolished the malignant phenotype induced by PRMT3, including proliferation, migration, and invasion in Huh7 cells (Fig. 3B–C). Furthermore, in Huh7 xenograft mouse model, JX06 treatment diminished the tumor-promoting effects of PRMT3 (Fig. 3D–F). Additionally, JX06 treatment blocked the increased p-PDHA and lactate levels induced by PRMT3 in the tumors (Fig. 3G–H, Supplementary Fig. S4C).

We then evaluated the PDHK1 role in Hepa1-6 cells and found that JX06 treatment also effectively eliminated the increased p-PDHA and lactate levels induced by Prmt3 (Supplementary Fig. S5A). To further explore whether the immune microenvironment affects the contribution of PDHK1 to PRMT3’s oncogenic role, we inoculated Hepa1-6 cells with or without Prmt3 overexpression into nude mice and immunocompetent C57BL/6 mice, respectively. Consistent with the results observed in the Huh7 cell subcutaneous model, Prmt3 overexpression increased tumor growth in both mouse strains. Notably, JX06 treatment effectively blocked the tumor-promoting effects of Prmt3 in nude mice, and exhibited a more pronounced inhibition in C57BL/6 mice (Supplementary Fig. S5B–E). These findings further confirmed the intrinsic role of PRMT3/PDHK1 axis in tumor promotion, and revealed that targeting PDHK1 exerts a more significant anti-tumor effect within the immune microenvironment.

### PRMT3 enhanced PD-L1 expression in tumor cells

**Fig. 4:**
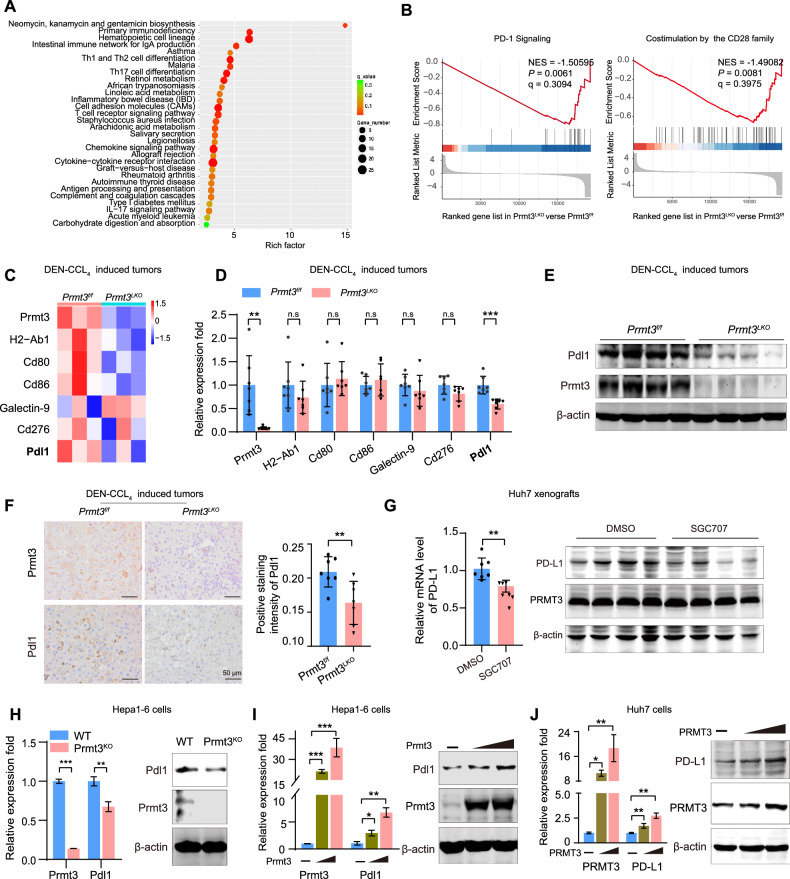
PRMT3 enhances PD-L1 expression in tumors. **A** KEGG analysis revealed the top 30 signaling pathways by utilizing RNA-seq data obtained from tumor tissues of *Prmt3*^*f/f*^ and *Prmt3*^*LKO*^ mice. *n* = 3 in each group for RNA-seq analysis. Differentially expressed genes were selected using a significance *q* value (*q* < 0.05), with a fold-change cut-off of 2 for upregulation and 0.5 for downregulation. **B** GSEA was performed for PD-1 Signaling and Co-stimulation by the CD28 family pathway using RNA-seq data. **C** A heatmap of tumor-associated immune checkpoint molecules expression profile based on GSEA. **D** Tumor-associated immune checkpoint molecules were detected by RT-PCR in tumor tissues of *Prmt3*^*f/f*^ and *Prmt3*^*LKO*^ mice. *n* = 7 in each group. **P < 0.01, ***P < 0.001, n.s, no significant difference. **E** Expression levels of Pdl1 were examined by immunoblotting in tumor tissues of *Prmt3*^*f/f*^ and *Prmt3*^*LKO*^ mice. **F** Expression levels of Prmt3 and Pdl1 determined using immunohistochemical staining. Scale bars, 50 μm. Right panel: Semi-quantitative analysis of Pdl1 positive staining in tumors. *n* = 7 in each group. **P < 0.01. **G** Expression of PD-L1 in Huh7 xenografts derived from nude mice treated with SGC707 was measured by RT-PCR (left) and immunoblotting (right). *n* = 7 in each group. **P < 0.01. **H** Expression of Pdl1 in Hepa1-6 cells with Prmt3 deletion was detected by RT-PCR (left) and immunoblotting (right). **P < 0.01. **I** Expression of Pdl1 in Hepa1-6 cells with Prmt3 overexpression were detected by RT-PCR (left) and immunoblotting (right). *P < 0.05, **P < 0.01, ***P < 0.001. **J** Expression of PD-L1 in Huh7 cells with PRMT3 overexpression was detected by RT-PCR (left) and immunoblot analysis (right). *P < 0.05, **P < 0.01. The data are presented as mean ± SD, and the statistical tests were all two-tailed.

To further investigate the effect of PRMT3 on tumor progression, we performed RNA-seq analysis on tumors isolated from *Prmt3*^*LKO*^ and *Prmt3*^*f/f*^ mice. Expression profile analysis revealed that 126 genes were upregulated and 534 genes were downregulated in *Prmt3*^*LKO*^ tissue compared to *Prmt3*^*f/f*^ tissue (Supplementary Fig. S6). Kyoto Encyclopedia of Genes and Genomes (KEGG) analysis revealed that Prmt3 deletion affects immune-related signaling pathways (Fig. 4A). Moreover, Gene Set Enrichment Analysis (GSEA) analysis revealed an enrichment of numerous genes involved in T cell-related signaling pathways, specifically the PD-1 signaling pathway and the B7 family interactions signaling pathway (Fig. 4B). Of note, a heatmap based on GSEA showed several tumor-associated immune checkpoints, such as *H2-Ab1, CD86, CD80, and CD276, CD274 (Pdl1)*, were downregulated upon Prmt3 deletion (Fig. 4C). We then validated the regulation of Prmt3 on these checkpoint molecules in tumor tissues. RT-PCR results demonstrated that *Pdl1* exhibited the most significant reduction among these checkpoint molecules in the tumors from *Prmt3*^*LKO*^ mice (Fig. 4D). Immunoblot and IHC staining also confirmed the reduction in Pdl1 protein levels in tumors from *Prmt3*^*LKO*^ mice (Fig. 4E–F). In Huh7 cell xenografts treated with SGC707, inhibition of PRMT3 activity also reduced PD-L1 expression (Fig. 4G). Additionally, knockout Prmt3 significantly decreased Pdl1 expression in Hepa1-6 cells at both mRNA and protein levels (Fig. 4H). Conversely, overexpression of PRMT3 promoted PD-L1 expression in both Hepa1-6 and Huh7 cells (Fig. 4I–J). Overall, these results suggest that PRMT3 modulates the tumor microenvironment by regulating PD-L1 expression.

### PRMT3 boosts PD-L1 expression by promoting histone lactylation modification

**Fig. 5:**
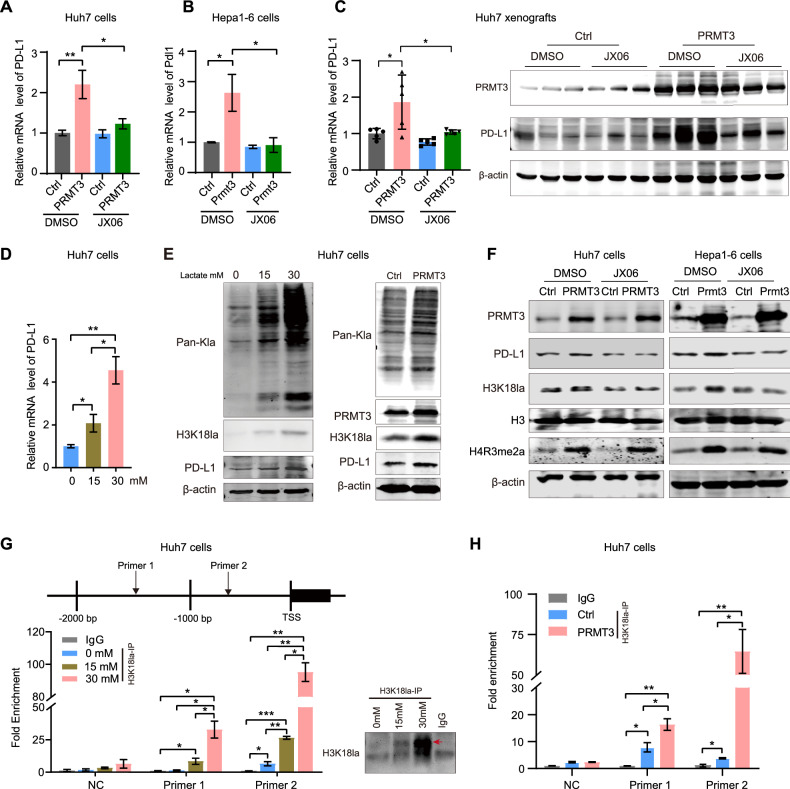
PRMT3 enhances PD-L1 expression through lactate-mediated histone lactylation. **A, B** *PD-L1* mRNA levels were measured in Huh7 and Hepa1-6 cells with the indicated treatments. Hepatoma cells were infected with Lenti-PRMT3 or Lenti-Ctrl and then treated with JX06 (200 nM) or DMSO. * *P* < 0.05, ** *P* < 0.01. **C** PD-L1 mRNA and protein levels were detected in Huh7 xenografts derived from nude mice with JX06 treatment. *n* = 5 in each group. *P < 0.05. **D** *PD-L1* expression in Huh7 cells was detected by RT-PCR after 24 hours of lactate stimulation at indicated concentrations. *P < 0.05, **P < 0.01. **E** PD-L1, pan-Kla, and H3K18la protein levels were detected by immunoblotting in Huh7 cells with lactate stimulation (left) or with PRMT3 overexpression (right). **F** Protein levels of PD-L1 and H3K18la were detected in Huh7 and Hepa1-6 cells with the indicated treatments. Briefly, Huh7 or Hepa1-6 cells pre-infected with lenti-PRMT3 or a control lentivirus (Ctrl) were seeded into 6-well plates at a concentration of 4 × 10^5^ cells per well for overnight culture. Then, these cells were treated with DMSO or JX06 (200 nM) for 24 hours to analyze protein levels by immunoblot. **G** Schematic diagram showing the primer site of the PD-L1 promoter for chromatin immunoprecipitation assay (upper). Huh7 cells were treated with indicated lactate concentration for 24 hours and subsequently subject to a chromatin immunoprecipitation assay with H3K18la antibody. ChIP assay revealed that lactate increased H3K18la occupancy at the PD-L1 promoter. Immunoblot analysis confirmed the immunoprecipitation of H3K18la. *P < 0.05, **P < 0.01, ***P < 0.001. **H** Enrichment of H3K18la at the PD-L1 promoter was detected in Huh7 cells with PRMT3 overexpression. *P < 0.05, **P < 0.01. The data are presented as mean ± SD, and the statistical tests were all two-tailed.

Next, we investigate whether PRMT3-induced glycolysis is involved in its promoting effect on PD-L1 expression. Notably, JX06 effectively blocked the effect of PRMT3 on PD-L1 expression in HCC cells and Huh7 xenografts (Fig. 5A–C). Moreover, the PD-L1 mRNA levels in Huh-7 cells increased upon different doses lactate stimulation, indicating that PRMT3 regulates PD-L1 expression in coordination with lactate level changes (Fig. 5D). Emerging evidence indicates that lactate-induced lactylation of histone H3 at lysine 18 (H3K18la) functions as a transactivation marker by enriching gene promoters and promoting gene expression in cancer cells. We found that both of lactate stimulation and PRMT3 overexpression elevated the levels of pan-Kla and H3K18la in Huh7 cells (Fig. 5E). Previous studies have demonstrated that PRMT3 may transactivate target genes by asymmetric methylating histone H4 at arginine 3 (H4R3me2a) [35]. Here, we found that PRMT3 enhances the levels of both H4R3me2a and H3K18la. However, JX06 treatment specifically blocked the elevation effect of PRMT3 on H3K18la and PD-L1 expression, but had no effect on H4R3me2a in either Huh7 or Hepa1-6 cells (Fig. 5F). This finding suggests that PRMT3 promotes PD-L1 expression not through methylation of histone H4 in this context. To further investigate the role of H3K18la in PD-L1 transactivation, we performed ChIP assays with H3K18la antibody in Huh7 cells. The results showed a significant increase in the enrichment of H3K18la at the PD-L1 promoter following lactate treatment, with higher lactate levels resulting in greater enrichment. (Fig. 5G). PRMT3 overexpression further increased H3K18la enrichment at the PD-L1 promoter (Fig. 5H). These findings indicate that PRMT3 promotes PD-L1 expression through promoting histone lactylation modification.

### Anti-PD-L1 blocked the oncogenic role of PRMT3 in HCC

**Fig. 6:**
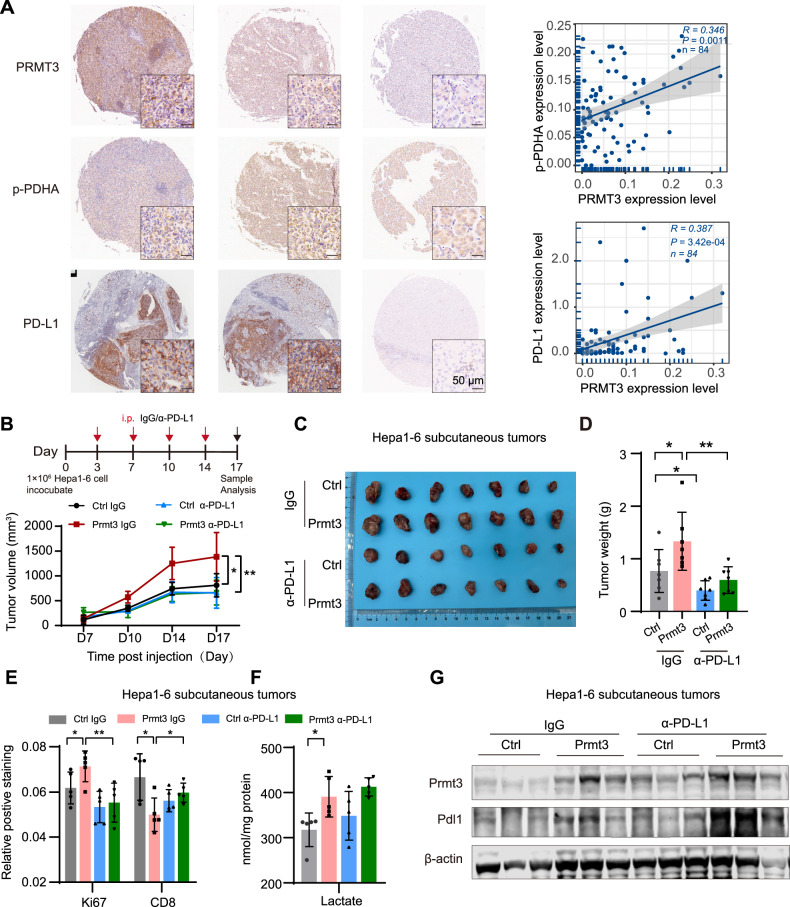
Anti-PD-L1 therapy blocks the tumor-promoting effects of PRMT3. **A** PRMT3, p-PDHA, and PD-L1 were immunohistochemically analyzed using a tissue microarray (TMA) of HCC specimens (left). Scale bar, 50 μm. TMA analysis revealed a positive correlation between PRMT3 and p-PDHA expression, as well as with PD-L1 expression (right). **B** Timeline of the anti-PD-L1 therapy in Hepa1-6 cells of the subcutaneous mouse model. Hepa1-6 cells infected with Lenti-Prmt3 or Lenti-Ctrl were inoculated subcutaneously in the right flank of C57/BL/6 mice. Anti-PD-L1 was administered intraperitoneally on day 3 post-inoculation (twice per week, 100 μg/mouse). Tumor growth curve was shown. *n* = 7 in each group, *P < 0.05, **P < 0.01. Tumor images (**C**) and tumor weight (**D**) were shown. *n* = 7 in each group, *P < 0.05, **P < 0.01. **E** Semi-quantitative analysis of Ki67 and CD8 positive staining in the indicated tumor groups. *n* = 5 in each group, *P < 0.05, **P < 0.01. **F** Lactate production was detected in the indicated tumor groups. *n* = 5 in each group, *P < 0.05. **G** PD-L1 expression levels in the indicated tumor groups were analyzed by immunoblotting. The data are presented as mean ± SD, and the statistical tests were all two-tailed.

We examined the protein expression levels of PRMT3, p-PDHA, and PD-L1 in HCC patient tissues using TMA analysis. The data revealed a positive correlation between PRMT3 expression and both p-PDHA and PD-L1 levels in tumor tissues (Fig. 6A). Furthermore, analysis of The Cancer Genome Atlas (TCGA) data showed a significant positive correlation between *PRMT3* and *PD-L1* expression in LIHC data (*r* = 0.357, *n* = 371, *P* < 0.001). Conversely, PRMT3 expression negatively correlated with CD8^+^ T cell infiltration in LIHC (*r* = -0.153, *n* = 371, *P* < 0.001) (Supplementary Fig. 7A). Notably, we also observed a significant positive correlation between *PRMT3* and *PD-L1* mRNA levels across various human cancer datasets from TCGA (Supplementary Fig. 7B).

We then investigated the effects of anti-PD-L1 treatment in HCC using subcutaneous models. As expected, Prmt3 overexpression significantly promoted tumor growth, increased tumor weight, and enhanced Ki67 staining, while markedly reducing CD8^+^ T cell infiltration. Anti-PD-L1 treatment effectively reduced tumor burden and restored CD8^+^ T cell infiltration (Fig. 6B–E, Supplementary Fig. 7C). Additionally, Prmt3 overexpression increased lactate accumulation and Pdl1 expression in tumors in IgG treatment group (Fig. 6F–G). Previous studies have demonstrated that immunotherapy can alter the transcriptional profile and microenvironment of tumors [36]. Consistent with this, we observed that the lactate accumulation and Pdl1 expression, which were originally regulated by Prmt3, were altered in the anti-PD-L1 therapy group. These findings suggest that anti-PD-L1 treatment counteracts the effects of PRMT3. Next, we analyzed patient cohorts with clinical data (GSE91061 for melanoma and ERP117672 for HCC) and found that *PRMT3* expression is significantly higher in the anti-PD-1 response group compared to the non-response group (Supplementary Fig. S7D–E). Collectively, our results indicating that PRMT3 may be a predictive biomarker for patients undergoing initial anti-PD-1/PD-L1 therapy.

## Discussion

Extensive research has firmly established a connection between the metabolism dysfunction and tumor immune evasion. In this study, we uncovered that PRMT3 promotes lactate accumulation by methylating PDHK1 at arginine 363/368 sites. Elevated lactate level in tumor facilitates histone lactylation, which is enriched at *PD-L1* promoter and ultimately enhanced *PD-L1* expression. Notably, anti-PD-L1 treatment abolished the oncogenic role of PRMT3 in HCC. Our findings unveil a novel metabolic-epigenetic regulatory axis that orchestrates tumor immunity in tumors.

Previous studies have demonstrated that PRMT3 promotes tumorigenesis by modifying various substrates. In pancreatic cancer, PRMT3 enhances chemoresistance by methylating heterogeneous nuclear ribonucleoprotein A1 (hnRNPA1), which stabilizes ABCG2 mRNA [24]. In endometrial carcinoma, PRMT3 drives malignant progression by methylating METTL14, reducing m^6^A modification of GPX4 [25]. In HCC, PRMT3 promotes glucose metabolism by methylating LDHA. Recent research has shown that PRMT3 methylates IGF2BP1 at R452, contributing to oxaliplatin resistance in liver cancer patients [37]. In this study, we identified PDHK1, a key regulator of tumor glycolytic adaptation, as a novel substrate of PRMT3 in HCC cells.

Pyruvate dehydrogenase kinases (PDKs) serve as a control switcher between glycolysis and oxidative phosphorylation (OXPHOS) by phosphorylating and inactivating its substrate, pyruvate dehydrogenase complex (PDC). This action prevents the conversion of pyruvate to acetyl-CoA, suppressing or even shutting down the OXPHOS, thereby promoting lactate accumulate. Notably, PDHK1 is frequently overexpressed in tumors, including HNSCC, melanoma, breast cancer, gastric cancer, and HCC, and is associated with poor patient prognosis [32, 38–41]. Indeed, numerous studies have shown that targeting PDHK1 reduces glycolysis, decreases lactate, increases ROS production, and leads to cell death, indicating its strong potential as a target for tumor therapy [42, 43]. Additionally, phosphorylation modifications at various serine, threonine, and tyrosine residues on the PDHK1 protein have been previously shown to influence its kinase activity and tumor progression [41, 44, 45]. Here, we report a distinct form of post-translational modification for PDHK1. We further demonstrated that PRMT3-mediated the methylation PDHK1 at arginine 363/368 sites, enhances its kinase activity, and thereby promoting lactate accumulation in cancer cells.

Lactate, the primary metabolite of glycolysis, is widely recognized as a key energy source driving tumor growth. Additionally, excessive lactate production acidifies the tumor microenvironment (TME), creating an immunosuppressive environment that hinders immune cell function. Lactate facilitates the infiltration of immunosuppressive cell types, such as M2 macrophages and regulatory T cells, thereby suppressing the antitumor immune response [46]. Moreover, lactate inhibits the cytolytic activity of CD8^+^ T cells and natural killer (NK) cells, contributing tumors in evading immune surveillance [47]. Our findings show a positive correlation between PRMT3 expression and lactate levels, and a negative correlation with CD8^+^ T cell infiltration in tumors. Furthermore, we observed that PRMT3-induced lactate increases PD-L1 expression in HCC cells, suggesting that lactate not only modulates immune cells but also enhances the immunosuppressive environment by upregulating PD-L1 expression in HCC cells.

Elevated PD-L1 on tumor cells interacts with PD-1 on T cells, suppressing T-cell response. PD-L1 is a critical, but not perfect, selective marker for stratifying patients for anti-PD-1/PD-L1 therapies in various tumors. Therefore, extensive research has focused on identifying the regulatory mechanisms of PD-L1 expression, including genomic, epigenetic and post-transcriptional factors, which may help to design new strategies to manipulate the PD-L1 expression to enhance immunotherapy efficacy [48–51]. Previous studies have showed that histone modification directly control PD-L1 expression. H3K4 trimethylation (H3K4me3) is enriched in the PD-L1 promoter and activates its transcription in pancreatic tumor cells [52]. HDAC inhibitors treatment could relax the chromatin state, enhancing acetylated histone 3 binding at the PD-L1 promoter, thereby upregulated the expression of PD-L1 in melanoma cells [53].

Lysine lactylation (Kla), a novel post-translational modification (PTM) derived from lactate, plays a crucial role in carcinogenesis [54–56]. Histone lactylation has emerged as a new epigenetic marker that stimulates gene expression [14, 57]. Particularly, recent studies have shown that PD-L1 expression can be epigenetically regulated by histone lactylation. In acute myeloid leukemia (AML), activated STAT5 promotes glycolysis, leading to lactate accumulation, which facilitates histone lactylation and enriches H4K5la at the PD-L1 promoter, enhancing its transcription [58]. In non-small cell lung cancer (NSCLC), H3K18la activates the transcription of POM121, promoting the binding of MYC to the PD-L1 promoter [59]. Additionally, integrative lactylome and proteome analyses have revealed that lactylation modifications are prevalent in HCC and contribute to tumor progression [60, 61]. Our current investigation shows that PRMT3-induced lactate accumulation increases H3K18la levels and epigenetically regulates the expression of PD-L1. We demonstrate that H3K18la acts as a direct epigenetic transactivator for PD-L1 in HCC. These findings illuminate the regulatory mechanisms of the PRMT3-PDHK1-lactate-PD-L1 axis and further establish the connection between metabolic byproducts and epigenetic regulation.

Immunotherapy has made significant strides in cancer treatment; however, the patient response rates remain relatively low. Metabolic dysfunction may partially explain the low immune response rate [8]. Most solid tumors exhibit features of metabolic adaptation to glycolysis and high lactate production [62–64]. Our analysis of pan-cancer data from TCGA reveals a positive correlation between PRMT3 and PD-L1 expression across various tumor types, suggesting that the PRMT3-lactate-PD-L1 regulatory axis may be a common mechanism in different cancers. Our study demonstrates that anti-PD-L1 therapy effectively counteracts the tumor-promoting effects of PRMT3 and restores CD8^+^ T cell infiltration in mouse tumor models. Furthermore, we analyzed public patient cohorts (GSE91061 for melanoma, ERP117672 for HCC) on anti-PD-1 treatment and found that PRMT3 expression is significantly higher in responders compared to non-responders [36, 65]. These findings indicate that PRMT3 could serve as a biomarker for patients undergoing initial anti-PD-1/PD-L1 therapy.

Monotherapy with either cytotoxic chemotherapy or immunotherapy often associates with considerable sides and the risk of drug or immune resistance in advanced HCC patients. Combination therapy is currently the mainstream in tumor therapy. Targeting PRMT3 with SGC707 treatment enhances oxaliplatin response in HCC, and combining SGC707 with anti-PD-1 therapy boosts antitumor effects in endometrial carcinoma [25, 37]. Similarly, targeting PDHK1 synergizes with other chemotherapeutics [66, 67]. Although the inhibitors targeting PDKs or PRMT3 have been developed, their efficacy remains to be clinically confirmed [68–70]. Future efforts should aim to develop more potent and specific small molecule inhibitors targeting PRMT3 or PDHK1, with minimal impact on normal cells. In addition, targeting the PRMT3-mediated methylation of PDHK1 to disrupt the PDHK/PDHA phosphorylation regulatory axis is a potential therapeutic strategy. Other glycolysis-lactate-regulated immune checkpoints may also be candidates for combination therapy with PRMT3 or PDHK1 inhibitors. A thorough understanding of the metabolic signature of tumors will support the development of more effective therapeutic interventions.

In summary, our study reveals that PRMT3 promotes glycolysis by activating PDHK1, which increases lactate accumulation and facilitates PD-L1 expression through enhanced the level of H3K18la. We present a novel metabolic-epigenetic regulatory axis that governs tumor immunity in tumors with high PRMT3 expression. Combining PRMT3 expression, tumoral lactate levels, and PD-L1 expression may inform personalized therapy strategies tailored to individual patients.

## Materials and methods

### Ethics approval and consent to participate

All methods employed in this study were conducted in accordance with the relevant guidelines and regulations. The animal care and experimental procedures complied with the National Institutes of Health Guide for the Care and Use of Laboratory Animals. This study obtained ethical approval from the Naval Medical University’s Ethical Review Committee and was acknowledged as projects supported by the National Natural Science Foundation of China (Grant No. 81802324, 82072641). For human tissue samples, all participants provided their written informed consent for the use of their tissue samples in scientific research.

### Human tissue samples

The specimens were collected from patients who underwent surgical resection and were diagnosed by specialized pathologists at the Eastern Hepatobiliary Surgery Hospital (Shanghai, China).

### Human Tumor Tissue Microarray (TMA)

Human hepatocellular carcinoma (LIHC) TMA was purchased from Shanghai Core Bio-Tech Co., Ltd (Shanghai, China). HlivH180Su16 for the analysis of PRMT3 and p-PDHA, and HlivH90Su01(a copy version containing all patients’ cancer tissues for HlivH180Su16) for the analysis of PD-L1. Two independent pathologists assessed immunohistochemistry staining. The staining intensity score ranged from 0 to 3, reflecting the percentage of immunoreactive staining area in the sample (0–10% was scored as 0, 11–25% as 1, 26–75% as 2, and 76–100% as 3). The final total score for each specimen was calculated by multiplying the staining intensity score by the staining extent score.

### Animals and treatment

*Prmt3*^f/f^ mice were established and purchased from Shanghai Model Organisms Center, Inc (Shanghai, China). Albumin-Cre (Alb-Cre) mice were obtained from Jackson Laboratory (Stock No. 003574, Maine, USA). Alb-cre; *Prmt3*^*f/f*^ (*Prmt3*^*LKO*^) mice were generated by crossing *Prmt3*^*f/f*^ mice with Alb-Cre mice. To induce primary HCC in mice, two-week-old *Prmt3*^*f/f*^ and *Prmt3*^*LKO*^ mice were administered with DEN (25 mg/kg, Sigma-Aldrich, St. Louis, USA) via intraperitoneal injection, followed by twice-weekly intraperitoneal injections of 10% CCl_4_ (dissolved in oil, 0.5 ml/kg, HY-Y0298, MCE, New Jersey, USA) at six weeks of age. The mice were then sacrificed at 18, 22, and 26 weeks of age.

For the subcutaneous injection model, five-week-old male athymic BALB/c nude mice were purchased from Shanghai BK/KY Biotechnology Company (Shanghai, China). Pretreated Huh7 cells (1 × 10^6^ per mouse) or pretreated Hepa1-6 cells were subcutaneously injected into the right flank of each mouse. When the tumors reached about 100 mm^3^ in size, mice were randomized into different groups.

Tumor-bearing mice in the corresponding subgroups were intraperitoneally administered JX06 (MedChemExpress, HY-19564, New Jersey, USA) at a 30 mg/kg dose every 2 days, or SGC707 (MedChemExpress, HY-19715, Shanghai, China) at a 30 mg/kg dose every 2 days, or vehicle control [71]. Both JX06 and SGC707 were dissolved in a solution containing 10%DMSO and 90% corn oil (MedChemExpress, HY-Y1888, New Jersey, USA) for used in vivo.

For immunotherapy experiments, five-week-old C57BL/6 mice were purchased from Shanghai Model Organisms Center, Inc. Pretreated Hepa1-6 cells (1×10^6^ per mouse) were injected into the right flank of these mice. After 3 days of subcutaneous injection, 100 μg/mouse anti-PD-L1 antibody (BioXCell, BE0101, New Hampshire, USA) or normal control IgG (BioXCell, BE0093, New Hampshire, USA) were intraperitoneally administered twice per week until the completion of the study. More detailed descriptions of the treatments are provided in the figure legends. Tumor volume was calculated using the following equation: volume = length×(width)^2^×1/2. All animals for this study were male.

### Multiplex immunofluorescences assay

For fluorescent multiplex IHC analysis, a four-color fluorescence kit (Novo-light, M-D110041, Shanghai, China) based on tyramide signal amplification (TSA) was used following the manufacturer’s protocol. The prepared tissue slides were deparaffinized and rehydrated using xylene and graded ethanol. Following incubation with 0.3% hydrogen peroxide and antigen retrieval in citrate buffer, the tissues were blocked with 5% BSA. Primary antibodies were added followed by TSA solution. After the last TSA cycle, the slides were incubated with a DAPI solution. The slides were then captured and scanned using K-viewer software from KFBIO technology. The primary antibodies included: anti-CD8 (Abcam, ab217344, 1:200, RRID: AB_2890649, Cambridge, United Kingdom), anti-PD-1 (Abcam, ab214421, 1:500, RRID: AB_2941806, Cambridge, United Kingdom), and anti-Granzyme B (Abcam, ab4059, 1:500, RRID: AB_304251, Cambridge, United Kingdom).

### Generation of knockout cell lines with CRISPR-Cas9

Hepa1-6 cells with *Prmt3* knockout were generated by Cyagen company (Shanghai, China) using CRISPR-Cas9 technology. The guide RNA sequence targeting mouse Prmt3 was GTCGGACAGCGGAGACGACG-CGG.

### Immunoprecipitation assay

Pretreated Huh7 cells were lysed with IP lysis buffer (Pierce, 87787, Illinois, USA) containing protease inhibitor. Cell lysates were centrifuged at 12000×g for 10 minutes. The supernatants were then incubated with the indicated primary antibody and protein G agarose beads (Sigma-Aldrich, 11243233001, St. Louis, USA) or incubated with the anti-Flag agarose beads with rotation at 4 °C overnight. On the second day, the beads were washed with a pre-cooled wash buffer. After centrifugation, the proteins were boiled for 10 minutes, and performed immunoblot analysis.

### Lactate measurement

The lactate levels in mouse tumor tissues or cultured cells under different treatments were measured using a Lactate Assay kit (Abcam, ab65331, Cambridge, United Kingdom), following the manufacturer’s protocol.

### Triglyceride (TG), total cholesterol (TC), and methionine levels measurement

TG, TC, and methionine levels in mouse tumor tissues were measured according to the manufacturer’s protocol. The TG Kit (mlbio, ml076635, Shanghai, China), the TC Kit (mlbio, ml076637, Shanghai, China), and the methionine ELISA kit (mlbio, ml077300, Shanghai, China) were used.

### Immunofluorescences assay

Cells were washed with PBS, fixed in 4% PFA, and permeabilized with 0.3% Triton X-100 in PBS for 5 min at room temperature, and subsequently incubated in blocking buffer (5% normal donkey serum in PBS). After an overnight incubation with primary antibodies at 4 °C, the cells were further incubated with Alexa Fluor 488- and Alexa Fluor 546-conjugated secondary antibodies (ThermoFisher Scientific, A11094 and A10036, Waltham, USA). The slides were then mounted using a DAPI-containing medium (Sigma-Aldrich, DUO82040, St. Louis, USA), and imaged using a Leica TCS SP5 confocal microscope. The antibodies used are listed in Table S2.

### Proximal ligation assay (PLA)

Pretreated cells were grown on coverslips and then fixed in 4% paraformaldehyde (PFA) for 15 minutes. Then the cells were washed, blocked, and incubated with primary antibodies at 4 °C overnight. Subsequently, the samples were washed and incubated with diluted PLUS® and MINUS® Duolink In Situ PLA Probes (1:5, Sigma-Aldrich, St. Louis, USA) at 37 °C for 1 hour. After three washes in Buffer A®, the cells were incubated in 1 unit/μl of T4 DNA ligase in diluted ligase buffer (1:5) for 30 minutes at 37 °C. Following this, the cells were washed three times in Buffer A®, and incubated in 5 units/μl of DNA polymerase in diluted polymerase buffer with green fluorescent dye-labeled oligonucleotides (1:5) for 100 minutes at 37 °C. Finally, the cells were washed with 1× Buffer B® for 10 minutes, followed by 0.01× Buffer B® for 1 minute. The slides were mounted in a 5 μL volume of DUOlink in situ mounting medium containing DAPI. Then the images were acquired using a Leica TCS SP8 confocal microscope.

### RNA-Seq

Total RNA was extracted from tumor tissues of *Prmt3*^f/f^ and *Prmt3*^*LKO*^ mice using TRIzol Reagent (Life Technologies, 15596026, Gaithersburg, MD, USA) according to the manufacturer’s instructions. The RNA quality was assessed with an Agilent 2100 Bioanalyzer, ensuring that all samples had an RNA integrity number (RIN) greater than 7 before proceeding with RNA sequencing library preparation. Sequencing was performed in pair-end 150 bp mode on an Illumina NovaSeq 6000. The reads were aligned to the GRCm38 reference genome using Hisat2 (version 2.04).

### Chromatin immunoprecipitation (ChIP) assay

Pretreated Huh7 cells were cross-linked with 1% formaldehyde for 15 minutes. A solution of 1.25 M glycine was used to terminate the cross-linking for 5 minutes. The cells were resuspended with ChIP lysis buffer (50 mM Hepes-KOH pH 7.5, 140 mM NaCl, 1 mM EDTA pH 8.0, 1% Triton X-100, 0.1% Sodium Deoxycholate, 0.1% SDS) and then sonicated to shear the DNA to an average fragment size of 200 to 1000 bp. Chromatin fragments were immunoprecipitated with an H3K18la antibody, and protein G agarose beads (Sigma-Aldrich, 11243233001, St. Louis, USA). DNA extraction was performed using glycogen, sodium acetate, and ethyl alcohol, and subsequently purified using Qiagen Purification Kits (QIAGEN, 28104, Duesseldorf, Germany). The enrichment of genes was analyzed by real-time PCR. The primer sequences for ChIP-PCR are listed in Table S3.

### Statistical analysis

All data were analyzed with Prism 8 (GraphPad Software, La Jolla, CA, RRID:SCR_002798).

The Levene test was conducted to assess the equality of variances among the continuous variables. Two-group datasets were analyzed using Student’s *t*-tests for normally distributed data, while the Mann-Whitney U test was applied for non-normally distributed data. One-way ANOVA was determined for the analysis of multiple groups involving a single variable. Two-way ANOVA was employed to compare more than two groups with multiple variables. The results are presented as the mean ± SD. All in vitro experiments were conducted in at least three independent experiments. Statistical tests were two-tailed, and *P* < 0.05 was considered statistically significant. Statistical significance was denoted as *P < 0.05, **P < 0.01, ***P < 0.001, and “n.s” indicates no significant difference.

## Supplementary information

supplement material

original western blots

## Data Availability

The RNA sequencing data are publicly available at Gene Expression Omnibus (GEO) under accession number GSE277664. All other data and materials supporting our findings can be made available from the corresponding authors upon request.
