# Supplementary material for: PRMT3 drives PD-L1-mediated immune escape through activating PDHK1-regulated glycolysis in hepatocellular carcinoma
Source: Cell Death Dis. 2025 Mar 6;16(1):158. doi: 10.1038/s41419-025-07482-7 (PMC11885674; doi:10.1038/s41419-025-07482-7)
Supplement: Supplementary file 1 — supplement material [file 41419_2025_7482_MOESM1_ESM.pdf]

Supplementary information for

PRMT3 drives PD-L1-mediated immune escape through activating PDHK1-regulated glycolysis in hepatocellular carcinoma

Correspondence to: zhang68@hotmail.com;

weifenxie@medmail.com.cn

Supplementary figures

Figure S1

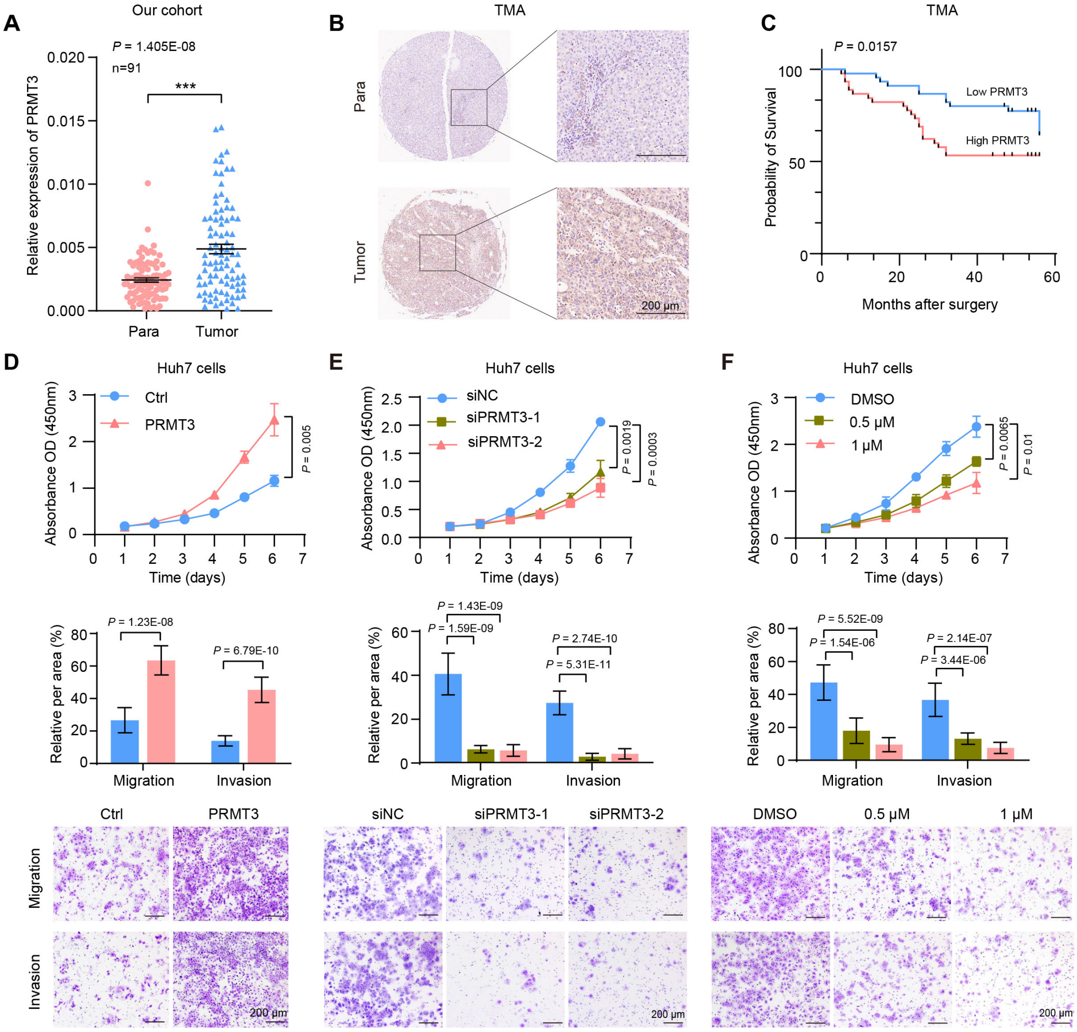

Figure S1. PRMT3 plays an oncogenic role in HCC

A, RT-PCR analysis of PRMT3 mRNA expression level in 91 pairs of HCC and corresponding adjacent non-tumor tissues. The *P*-value for comparison between para-tumor tissue and tumor tissue was < 0.001. The *P* value was analyzed using non-parametric Mann–Whitney test. **B,** Representative immunohistochemistry staining images of PRMT3 in HCC and adjacent non-tumor tissues from tissue microarray patients (TMA). Scale bar, 200 μm. **C,** Overall survival of patients in TMA according to the expression of PRMT3 in tumors (n = 90). The median value of PRMT3 expression was used as the cut-off to separate the patients. Using the log-rank test, the *P*-value for the comparison between patients with high PRMT3 expression and those with low PRMT3 expression was < 0.05. **D,** Huh7 cells were infected with lenti-PRMT3 or control lentivirus (Ctrl), and then the proliferation, migration and invasion capacity were measured. The representative images of stained transwell chamber were shown at the bottom. Scale bar, 200 μm. **E,** Huh7 cells were transfected with siPRMT3 or siNC, and then the proliferation, migration and invasion capacity were measured. The representative images of stained transwell chamber were shown at the bottom. Scale bar, 200 μm. **F,** Huh7 cells were treated with SGC707 at the indicated concentration or DMSO, then the proliferation capacity, migration and invasion capacity were measured. The representative images of stained transwell chamber were shown at the bottom. Scale bar, 200 μm. The data are presented as mean ± SD, and the statistical tests were all two-tailed.

Figure S2

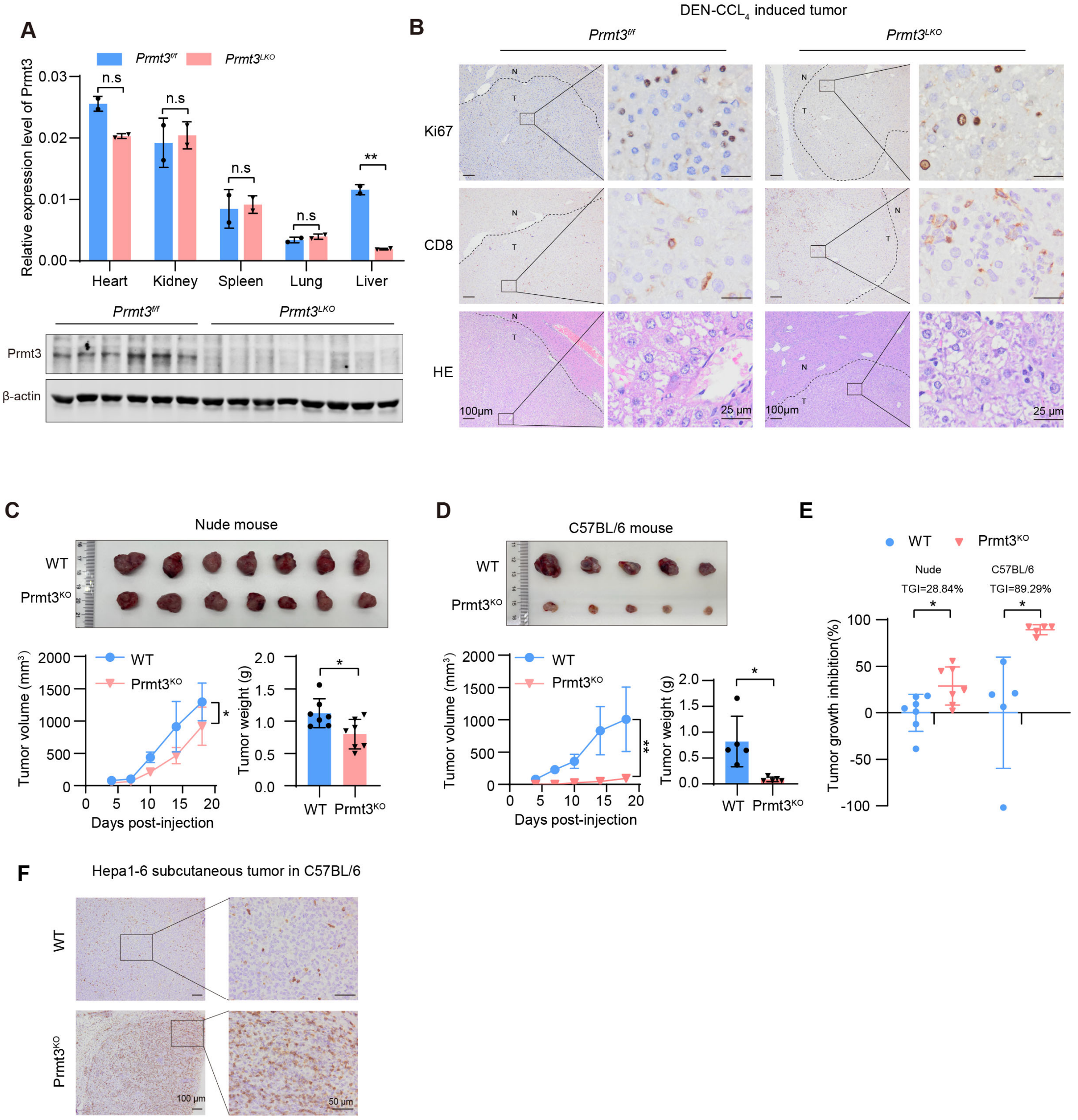

Figure S2. *Prmt3* deletion inhibits tumor growth

A, Validation that the *Prmt3* was specifically knocked out in livers of *Prmt3*^{LKO} mice via RT-PCR (upper) and immunoblot (lower). n = 2 in each group for RT-PCR test. **B,** Representative images of immunohistochemical staining of Ki67, CD8, and HE in tumor tissues of *Prmt3*$^{f/f}$ and *Prmt3*^{LKO} mice at 26 weeks. Scale bar, 25 μm or 100 μm. The magnification used for the images is either 100-fold or 1000-fold. **C,** Tumor gross appearance, growth curve, and weight of Prmt3 knockout (Prmt3^{KO}) and untreated Hepa1-6 cells (WT) implanted into nude mice. n =

7 in each group. **D,** Tumor gross appearance, growth curve, and weight of Prmt3^{KO} and WT Hepa1-6 cells implanted into immunocompetent C57BL/6 mice. $n = 5$ in each group. **E,** Tumor growth inhibition assessed in nude mice and C57BL/6 mice. The central line indicated the mean value. $n = 5$ in each group. **F,** Representative images of CD8 staining in Hepa1-6 tumors derived from C57BL/6 mice. Scale bar, 50 μm. The magnification used for the immunohistochemical staining is either 100-fold or 400-fold. * $P < 0.05$, n.s, no significant difference. The data are presented as mean ± SD, and the statistical tests were all two-tailed.

Figure S3

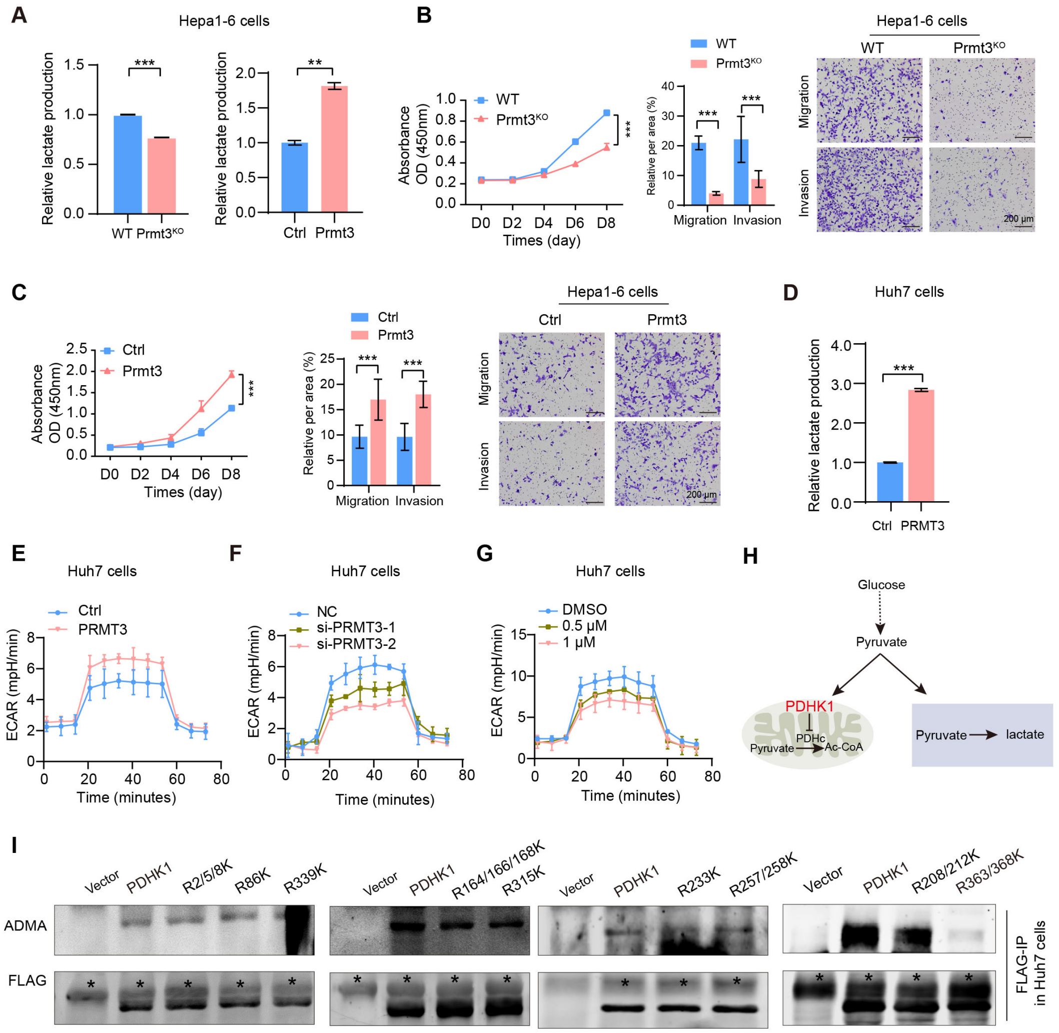

Figure S3. PRMT3 promotes lactate accumulation by methylating PDHK1 in hepatoma cells

A, Lactate levels were measured in Hepa1-6 cells with Prmt3 knockout or with Prmt3 overexpression. **B,** The proliferation capacity, migration and invasion capacity were measured in Hepa1-6 cells (WT) and Prmt3 knockout Hepa1-6 cells. The stained images of transwell chamber were represented at the right corner. **C,** Hepa1-6 cells were infected with lenti-Prmt3 or control lentivirus (Ctrl), and then the proliferation, migration and invasion capacity were measured. The stained images of transwell chamber were represented at the right corner. **D,** Lactate levels were measured in Huh7 cells with PRMT3 overexpression. **E,** Glycolytic function of Huh7 cells infected with lenti-PRMT3 or control lentivirus (Ctrl) was detected. **F-G**, The glycolytic function of Huh7 cells transfected with siPRMT3 (F) or treated with SGC707 (G) was detected. **H,** A schematic diagram of PDHK1 function in the glycolysis process. **I,** Wildtype PDHK1, a series mutation of PDHK1, and their corresponding vector control were transfected into Huh7 cells with PRMT3 overexpression. Subsequently, these PDHK1 variants were immunoprecipitated using Anti-FLAG beads and subjected to immunoblot analysis to detect the level of ADMA. "*" in figure S3I indicated heavy chain. *** $P < 0.001$. The data are presented as mean ± SD, and the statistical tests were all two-tailed.

Figure S4

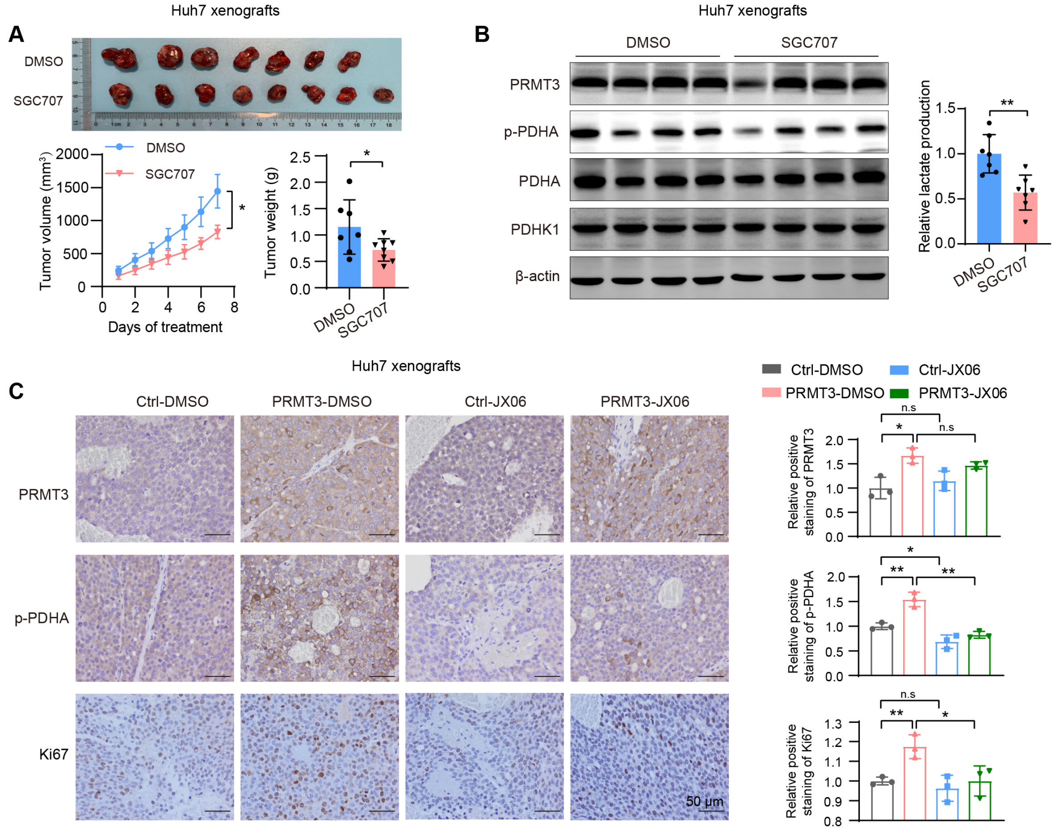

Figure S4. The methylation of PDHK1 mediated by PRMT3 enhances its kinase activity

A, Huh7 cells were implanted into nude mice. After subcutaneous tumor formation, the nude mice were administrated SGC707 or vehicle via intraperitoneal injection every 2 days at a dose of 30 mg/kg. Tumor image, growth curve and tumor weight of the Huh7 xenografts were evaluated. *n* = 7 or 8 in each group. **B,** Immunoblot analysis of PRMT3 and p-PDHA levels in Huh7 xenografts (left). Lactate levels were detected in Huh7 xenografts (right). *n* = 7 in each group for lactate test. **C,** Representative images of PRMT3, p-PDHA, and Ki67 staining in Huh7 xenografts with indicated treatments. Right panel: Semi-quantitative analysis of PRMT3, p-PDHA, and Ki67 staining in tumors. *n* = 3 in each group. Scale bar, 50 μm. The magnification used for the immunohistochemical staining is 400-fold. * $P < 0.05$, ** $P < 0.01$, n.s, no

significant difference. The data are presented as mean ± SD, and the statistical tests were all two-tailed.

Figure S5

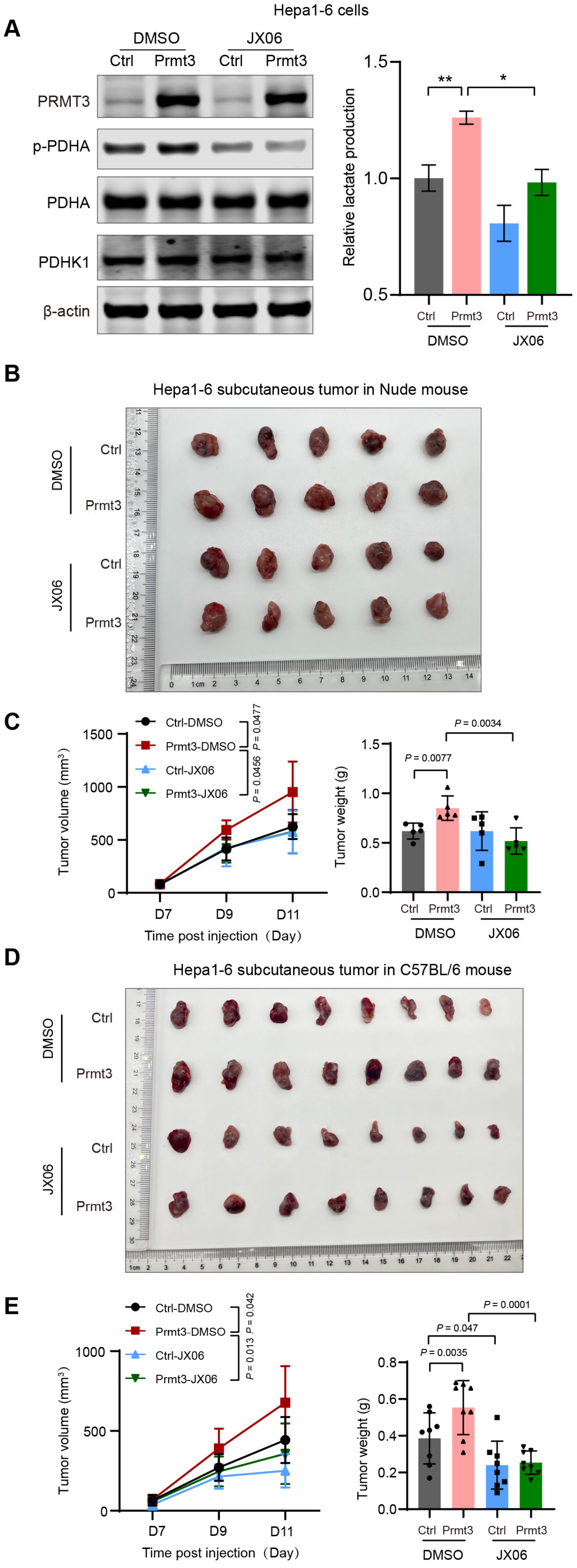
A
Hepa1-6 cells
DMSO
JX06
Ctrl
Prmt3
PRMT3
p-PDHA
PDHA
PDHK1
β-actin
Relative lactate production
**
*
B
Hepa1-6 subcutaneous tumor in Nude mouse
C
Tumor volume (mm3)
Ctrl-DMSO
Prmt3-DMSO
Ctrl-JX06
Prmt3-JX06
P = 0.0477
P = 0.0456
D7
D9
D11
Time post injection（Day)
Tumor weight (g)
P = 0.0077
P = 0.0034
D
Hepa1-6 subcutaneous tumor in C57BL/6 mouse
E
P = 0.042
P = 0.013
P = 0.0035
P = 0.047
P = 0.0001

Figure S5. PDHK1 contributes to the oncogenic role of Prmt3 in Hepa1-6 cells

A, Hepa1-6 cells pre-infected with lenti-Prmt3 or lenti-Ctrl were seeded into 6-well plates at a concentration of 4×10^5 cells per well for overnight culture. Then, these cells were treated with DMSO or JX06 (200 nM) for 24 hours and subject to immunoblot analysis or lactate detection. * $P < 0.05$, ** $P < 0.01$. **B-C,** Hepa1-6 cells infected with Lenti-Prmt3 or Lenti-Ctrl were inoculated into nude mice. On day 7 post-inoculation, tumor-bearing mice were administered JX06 or vehicle via intraperitoneal injection every 2 days at a dose of 30 mg/kg. Tumor image(B), growth curve, and tumor weight (C) of the Hepa1-6 tumors were evaluated. $n = 5$ in each group. **D-E,** Hepa1-6 cells infected with Lenti-Prmt3 or Lenti-Ctrl were inoculated into immunocompetent C57BL/6 mice. JX06 or vehicle was administered intraperitoneally on day 7 post-inoculation (every 2 days, 30 mg/kg). Tumor image(D), growth curve, and tumor weight (E) of the Hepa1-6 tumors were evaluated. $n = 8$ in each group. The data are presented as mean ± SD, and the statistical tests were all two-tailed.

Figure S6

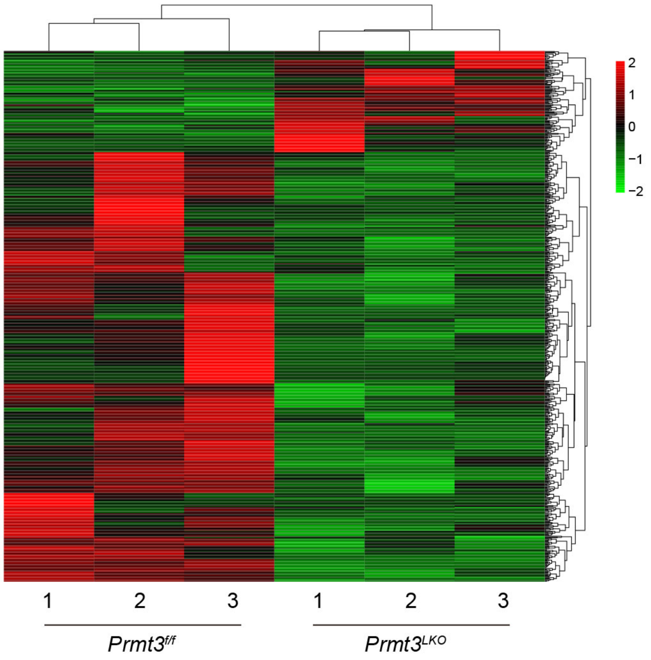

Figure S6. The heatmap depicts the profile of differentially expressed genes based on RNA-seq data obtained from *Prmt3$^{f/f}$* and *Prmt3^{LKO}* mice tumor tissue. The criteria for differential expression included a fold-change cut-off of 2 for upregulation and 0.5 for downregulation and a significant q value (q < 0.05).

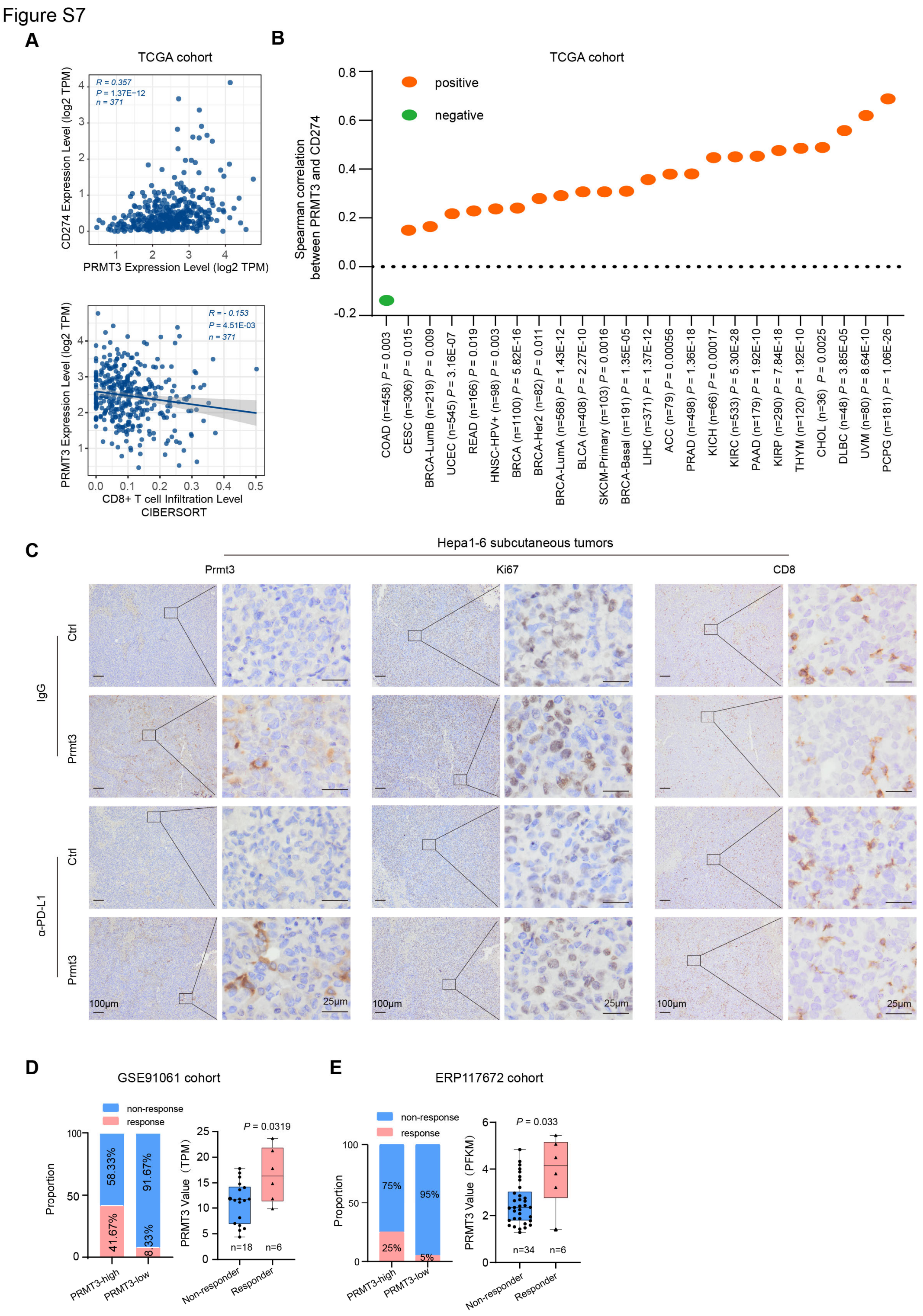

Figure S7. PRMT3 expression positively correlates with PD-L1

A, Expression levels of PRMT3 are positively correlated with expression levels of PD-L1, while inversely correlated with CD8^{+} T cell infiltration levels in LIHC from the TCGA database.

B, Correlation analysis of *PRMT3* and *PD-L1* in pan-cancer from the TCGA database. Spearman's correlation test was performed and *P* values < 0.05 in cancers were shown. **C,** Representative images of Ki67 and CD8 staining in the Hepa1-6 cells of the subcutaneous mouse model. Scale bar, 100 μm or 25μm. The magnification used for the immunohistochemical staining is either 100-fold or 1000-fold. **D**, The percentage of melanoma patients with response and non-response to anti-PD-1 therapy from GSE91061 cohort (left). Boxplot showing the PRMT3 expression level in anti-PD-1 responders and non-responders (right). The center values indicated the mean value. The RNA sequencing data, obtained from 24 naive patients (with no prior immunotherapy) before anti-PD-1 therapy from the GSE91061 dataset, was utilized to analyze the treatment response. The *P* value was analyzed using non-parametric Mann–Whitney test. **E,** The percentage of HCC patients with response and non-response to anti-PD-1 therapy from ERP117672 dataset (left). Boxplot showing the PRMT3 expression level in anti-PD-L1 responders and non-responders(right). The center values indicated the mean value. The RNA sequencing data, obtained from 40 pre-treatment HCC samples from ERP117672 dataset, were utilized to analyze the treatment response. The *P* value was analyzed using non-parametric Mann–Whitney test.

Supplementary materials and methods

Cell culture

The human HCC cell line Huh7 and mouse HCC cell line Hepal-6 were sourced from the Type Culture Collection of the Chinese Academy of Sciences (Shanghai, China) and validated using short tandem repeat analysis. Huh7 and Hepal-6 cells were cultured in Dulbecco’s modified

Eagle's medium (DMEM) containing 10% heat-inactivated fetal bovine serum (FBS, GIBCO, 10099141C, Brazil), under standard conditions (37 ℃, 5% CO_2). Mycoplasma contamination was routinely checked using the Mycoalert detection kit (Lonza, LT07-318, Basel, Switzerland).

Lentivirus

The coding sequences of human and mouse PRMT3 were individually subcloned into the expression vector pCDH-CMV-MCS-EF1-copGFP (System Biosciences, California, USA). Lentivirus was produced by co-transfecting 293T cells with this expression vector, along with packaging vector psPAX2 (Addgene, 12260) and envelope vector pMD2.G (Addgene, 12259), using Lipofectamine™ 2000 (Thermo Fisher Scientific, 11668019, Waltham, USA) according to the manufacturer's instructions. The supernatant, collected at 48- and 72-hours post-transfection, was centrifuged at 4 ℃ to remove cellular debris. The viruses were used immediately or stored at -80 ℃.

Immunoblotting assay

Protein lysates were collected from pretreated cells or mouse tissues using lysis buffer (125 mM Tris-HCl, pH 6.8, 25% glycerol, 5% SDS) containing the protease inhibitor PMSF (Beyotime, ST506, Shanghai, China). Protein concentrations were determined using the BCA Protein Assay (Beyotime, P0010, Shanghai, China). Samples were separated by SDS-PAGE and transferred to nitrocellulose membranes (Millipore, HAHY00010, Billerica, MA, USA). Membranes were blocked with 5% milk in PBST and incubated overnight at 4 ℃ with primary antibodies. After washing, membranes were incubated with secondary antibodies (donkey anti-mouse or donkey anti-rabbit, IRDye680/800) for 1 hour at room temperature. Detection was performed with the Odyssey Infrared Imaging System (LI-COR) at 700 nm or 800 nm. The

primary antibodies are listed in Table S2, Chemical reagents and antibodies used in this paper.

Histology and immunohistochemical analysis

Immunohistochemistry (IHC) staining was performed on 4 μm paraffin-embedded tumor tissue from mice. Staining was analyzed under a photomicroscope, and positive staining areas were quantified using Image-Pro Plus 6.0 software (Media Cybernetics, RRID:SCR_007369). The antibodies used in IHC staining are listed in Table S2, Chemical reagents and antibodies used in this paper.

RNA isolation and real-time PCR

Total RNA was extracted from tissues or cultured cells using TRIzol reagent (Takara, Tokyo, Japan). First-strand cDNA was synthesized using M-MLV Reverse Transcriptase (Promega, Madison, WI, USA). SYBR Green PCR Kit (Applied Biosystems, Waltham, USA) was used for RT-PCR, and β-actin served as the reference gene for normalization. Primer sequences are listed in Table S3, Primers and siRNA used in this paper.

Mass spectrometry

Huh7 cells pre-infected with PRMT3 lentivirus were transfected with FLAG-tagged PDHK1 for 24 hours. Then the cells were subjected to immunoprecipitation using FLAG-beads (Sigma-Aldrich, A7345, St. Louis, USA). The immunoprecipitants were washed three times with wash buffer (20 mM Tris [pH 8.0], 150 mM NaCl, 0.5% NP40, 0.5% TritonX-100, and 10% glycerol), followed by two washes with PBS buffer. The proteins bound to the beads were then subjected to trypsin digestion in an appropriate buffer. The resultant tryptic peptides were then examined using a Q ExactiveTM HF-X mass spectrometer (Thermo Scientific) for mass spectrometric analysis.

Gene overexpression and silencing

The full-length PRMT3 and PDHK1 genes or a series mutation of PDHK1 were synthesized by You-bio technology company (Changsha, China) and cloned into expression vectors. PRMT3 knockdown was achieved using siRNA transfected into HCC cells with Lipofectamine 2000 (ThermoFisher Scientific, 11668019, Waltham, USA), following the manufacturer's protocol. PRMT3-targeting siRNAs and the negative control (NC) were synthesized by GenePharma (Shanghai, China). The sequences of siRNA are listed in Table S3, Primers and siRNA used in this paper.

Cell viability

Cell viability was measured using the Cell Counting Kit-8 (CCK-8, Dojindo, CK04, Kumamoto, Japan) according to the manufacturer's protocol. HCC cells (3×10^3 per well) were seeded into 96-well plates and cultured in medium containing DMSO, 0.5 μM or 1 μM SGC707 to detect the effect of SGC707 on tumor cell growth. Similarly, Huh7 cells pre-infected with lenti-PRMT3 or a control lentivirus (Ctrl) were seeded into 96-well plates (3×10^3 cells per well) and cultured in medium containing DMSO or 200 nM JX06 to access the JX06 role on the proliferation of Huh7 cells. The medium containing the inhibitors was changed every two days. At the indicated times, 10% CCK-8 reagent was added and incubated for 1 hour at 37 °C. Absorbance was measured at 450 nm.

Migration and invasion assay

Cell migration and invasion assays were conducted using Transwell chambers (Falcon, 353097, New York, USA), with or without Matrigel (Corning, 354234, New York, USA). For migration, 5×10^4 pretreated cells were seeded in a serum-free medium into the upper chamber; for

invasion, 6 × 10^4 cells were used. The lower chamber contained medium with 10% FBS. To assess the effect of inhibitors on the migration and invasion abilities of tumor cells, serum-free medium containing 0.5 μM, 1 μM SGC707 or 200 nM JX06 was used in the upper chamber. The medium in the lower chamber contained the same concentrations of inhibitors and 10% FBS. After 48 hours of incubation at 37 °C, cells on the membrane's lower surface were fixed, stained with crystal violet, photographed, and quantified by counting in five random fields. Stained areas were measured with Image-Pro Plus 6.0 (Media Cybernetics, RRID:SCR_007369). Each experiment was repeated at least three times.

Glycolytic function test

The glycolytic function in HCC cells was analyzed using Seahorse XF Glycolytic Rate Assay Kit (Agilent Technologies, 103344, Santa Clara, CA, USA), following the manufacturer's instructions. Briefly, Huh7 cells were infected with Lenti-PRMT3, transfected with siPRMT3 for 24 hours, or treated with 0.5 μM or 1 μM SGC707 for 48 hours; afterwards, the pretreated Huh7 cells (8×10^3 per well) were plated in a 96-well assay plate for overnight culture. The following day, glycolytic function tests were conducted on a Seahorse XFe96 Analyzer (Agilent Technologies, RRID:SCR_019545).

Supplementary Tables

Supplementary Table 1: The peptides identified by MS.

Peptides sequence identified by MS	Modifications	Arginine Site	Dimethyl (KR)	Methyl (KR)	Oxidation (M)
GAALAGPGPGLR	Unmodified	20	0	0	0
SFSSDSGSSPASER	Unmodified	40	0	0	0
GVPGQVDFYAR	Unmodified	51	0	0	0
TSFMFLR	Unmodified	80	0	0	0
EISLLPDNLLR	Unmodified	103	0	0	0
AIYERPR	Unmodified	138/140	0	0	0
MIFTDTVIR	Oxidation (M)	/	0	0	1
MIFTDTVIR	Unmodified	164	0	0	0
ESFGVDPVTSQNVQYFLDR	Unmodified	203	0	0	0
DGYENAR	Unmodified	257	0	0	0
GGGVPLR	Unmodified	346	0	0	0
GGGVPLRK	Unmodified	346	0	0	0
LFNYMYSTAPR	Unmodified	361	0	0	0
AVPLAGFGYGLPISR	Unmodified	383	0	0	0
HYNTNHEADDWCVPSR	Unmodified	445	0	0	0
ALSTDSIER	Unmodified	419	0	0	0
DMTTFR	Unmodified	454	0	0	0

Supplementary Table 2. Chemical reagents and antibodies used in this paper.

Regents	Source	Cat No.	RRID
SGC707	MedChemExpress	HY-19715	N/A
JX06	MedChemExpress	HY-19564	N/A
Lactate	MedChemExpress	HY-B2227	N/A
Anti-FLAG M2 Affinity Gel	Sigma-Aldrich	A2220	AB_10063035
Anti-β-actin	Sigma-Aldrich	A5441	AB_476744
Anti-PD-L1	Proteintech	66248-1	AB_2756526
Anti-PRMT3	Abclonal	A13068	AB_2759916
Anti-PRMT3	Abcam	ab191562	N/A
Anti-Ki67	Servicebio	GB121141	AB_3083641
Anti-CD8	Abclonal	A22219	N/A
Anti-PDHK1	Abcam	ab110025	AB_10865315
Anti-PDHK1	CST	CST3820	N/A
Anti-ADMA	CST	CST13522S	N/A
Anti-PDHA	Santa cruz	sc-377092	N/A
Anti-p-PDHA	Abcam	ab177461	N/A
Anti-pan Kla	PTM	PTM-1401RM	N/A
Anti-H3K18la	PTM	PTM-1427RM	N/A
Anti-H4R3me2a	Active Motif	39006	AB_2793313
Anti-PD-L1 in vivo	BioXCell	BE0101	N/A
Anti-IgG in vivo	BioXCell	BE0093	N/A

Supplementary Table 3. Primers and siRNA used in this paper.

Gene	Forward primer (5’- 3’)	Reverse primer (5’- 3’)
β-actin(H)	CATCCTGCGTCTGGACCT	GTACTTGCGCTCAGGAGGAG
β-actin(m)	GTGACGTTGACATCCGTAAAGA	GCCGGACTCATCGTACTCC
PRMT3(H)	TACCCTTCTCATACCCCAA	CCCAACATCCAAAACTACC
Prmt3(m)	CTTGGGAAAAAGATGAGTA	GGGGTATGAGAATGGAGTA
H2-Ab1(m)	GAATGGGGACTGGACCTTC	TTTCTGACTCCTGTGACGG
Cd80(m)	TGACTTCTCTACCCCCAAC	TCTCTTCCATTTTCCAACC
Cd86(m)	CCCGAAACCTAAGAAGATG	TTCCAGAACACACACAACG
Galentin-9(m)	CTCTTCAGTGCCCAGTCTC	TGTCCGTTCTGCTTCGTGT
Cd276(m)	AGGGTGGTGCTGGGTGCTA	GCCTCAGGGGGGAATGTCA
PD-L1(m)	GTACGTGGTGGAGTATGGC	CTCTGGTTGATTTTGCGGT
PD-L1(H)	GCTCCAAAGGACTTGTACGTG	TGATCTGAAGGGCAGCATTTC
Negative for CHIP	ACCAACACTCTTCCCTCAGC	TTATTTTGGTTCAGGTGGTTGA
PD-L1-P1	GCTGCTGACTTTTTATAT	CTAGTGTTGGTGTCCTAG
PD-L1-P2	GTCAGGAAAGTCCAACGCC	CTCCATCCCAAAGAAAGGG
NC (siRNAs)	UUCUCCGAACGUGUCACGUtt	ACGUGACACGUUCGGAGAAtt
siPRMT3#1	CCUUGGGAGAAAGAAGAGAUtt	AUCUCUUCUUUCUCCCAAGGtt
siPRMT3#2	CCUUGUGGUAUUAAGCAUAUAtt	UAUAUGCUUAAUACCACAAGGtt
