# Supplementary material for: PRMT3 drives PD-L1-mediated immune escape through activating PDHK1-regulated glycolysis in hepatocellular carcinoma
Source: Cell Death Dis. 2025 Mar 6;16(1):158. doi: 10.1038/s41419-025-07482-7 (PMC11885674; doi:10.1038/s41419-025-07482-7)

Original figures for western blots in this paper

Figure 2


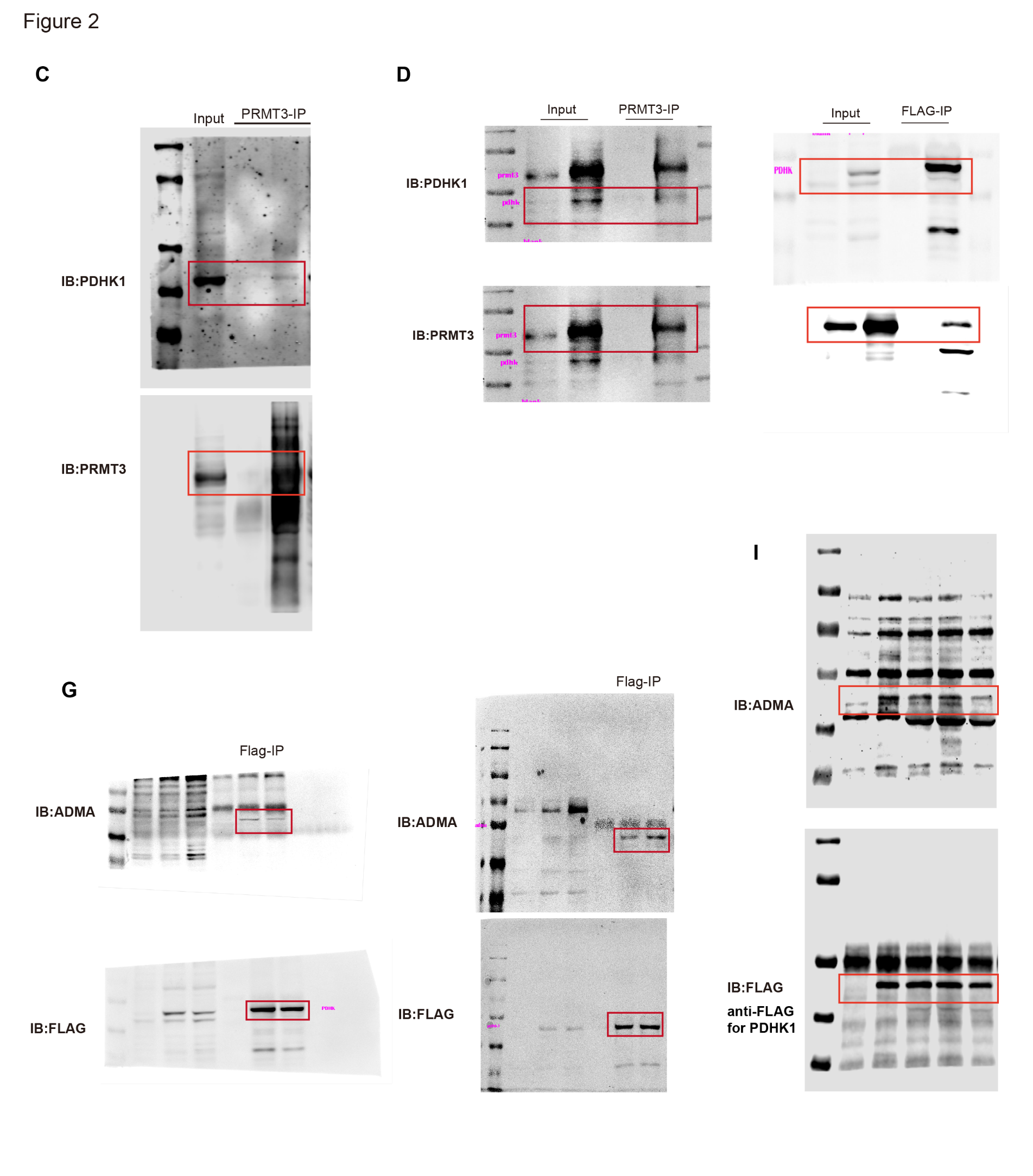


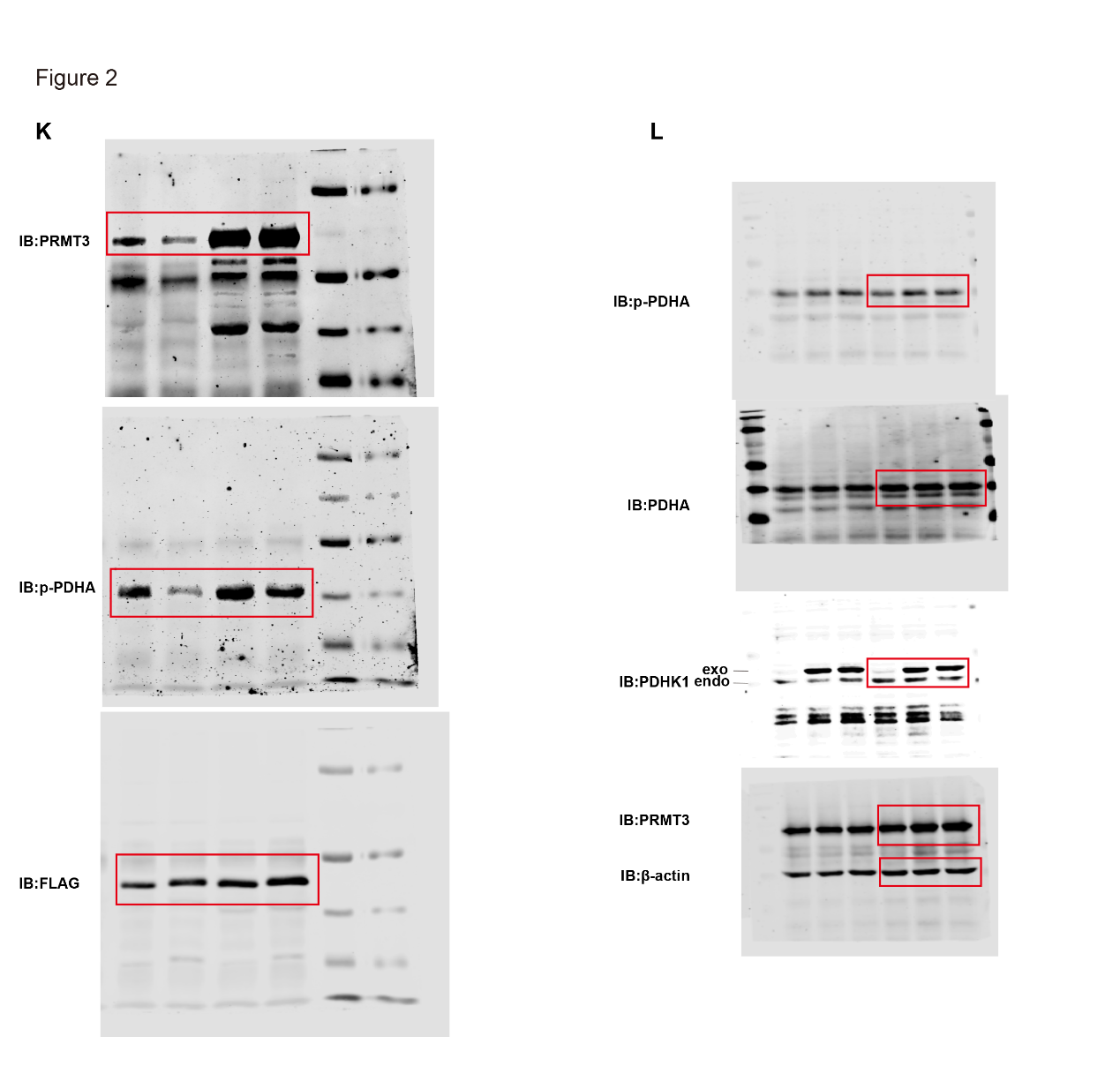


Figure 3


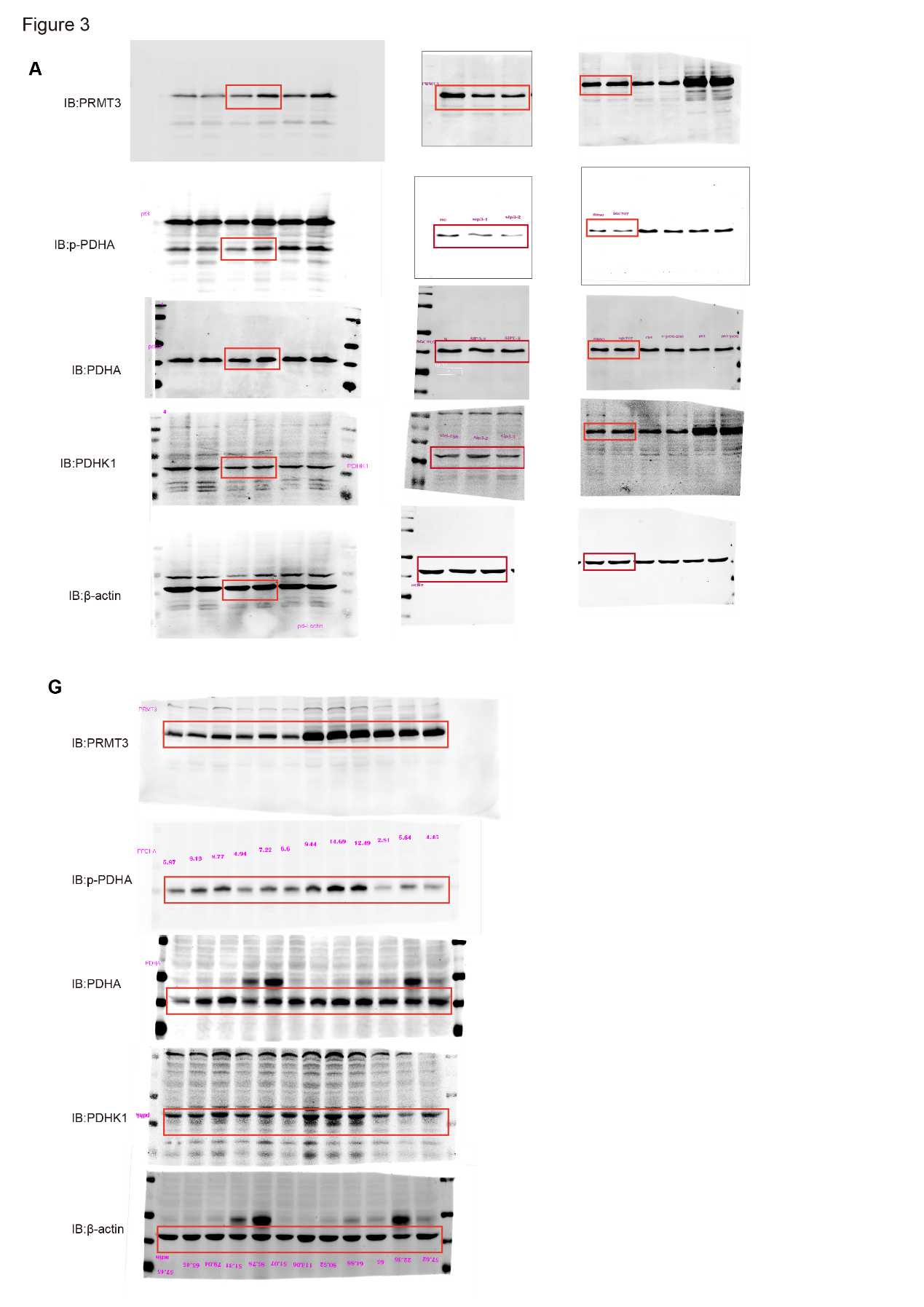


Figure 4


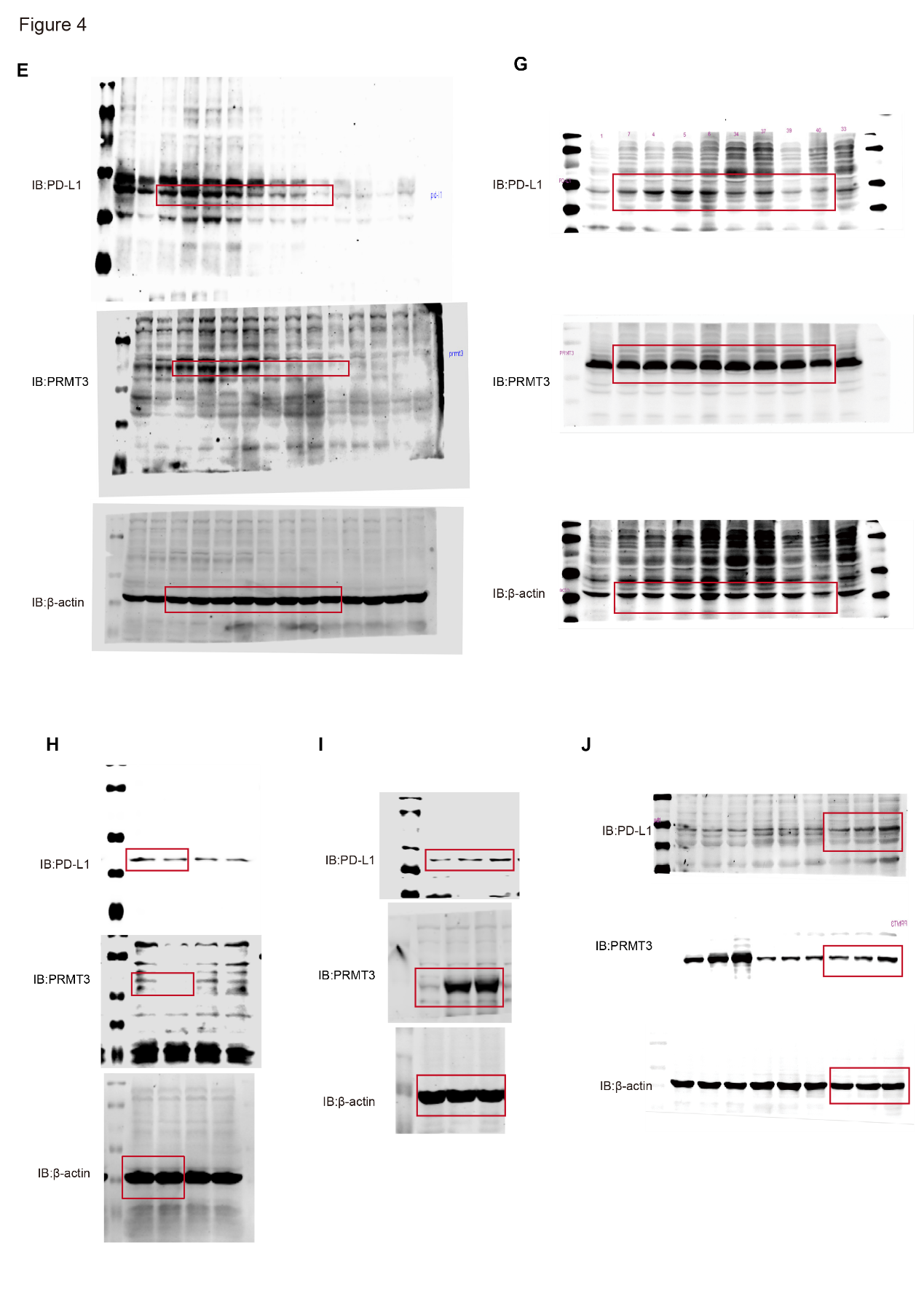


Figure 5
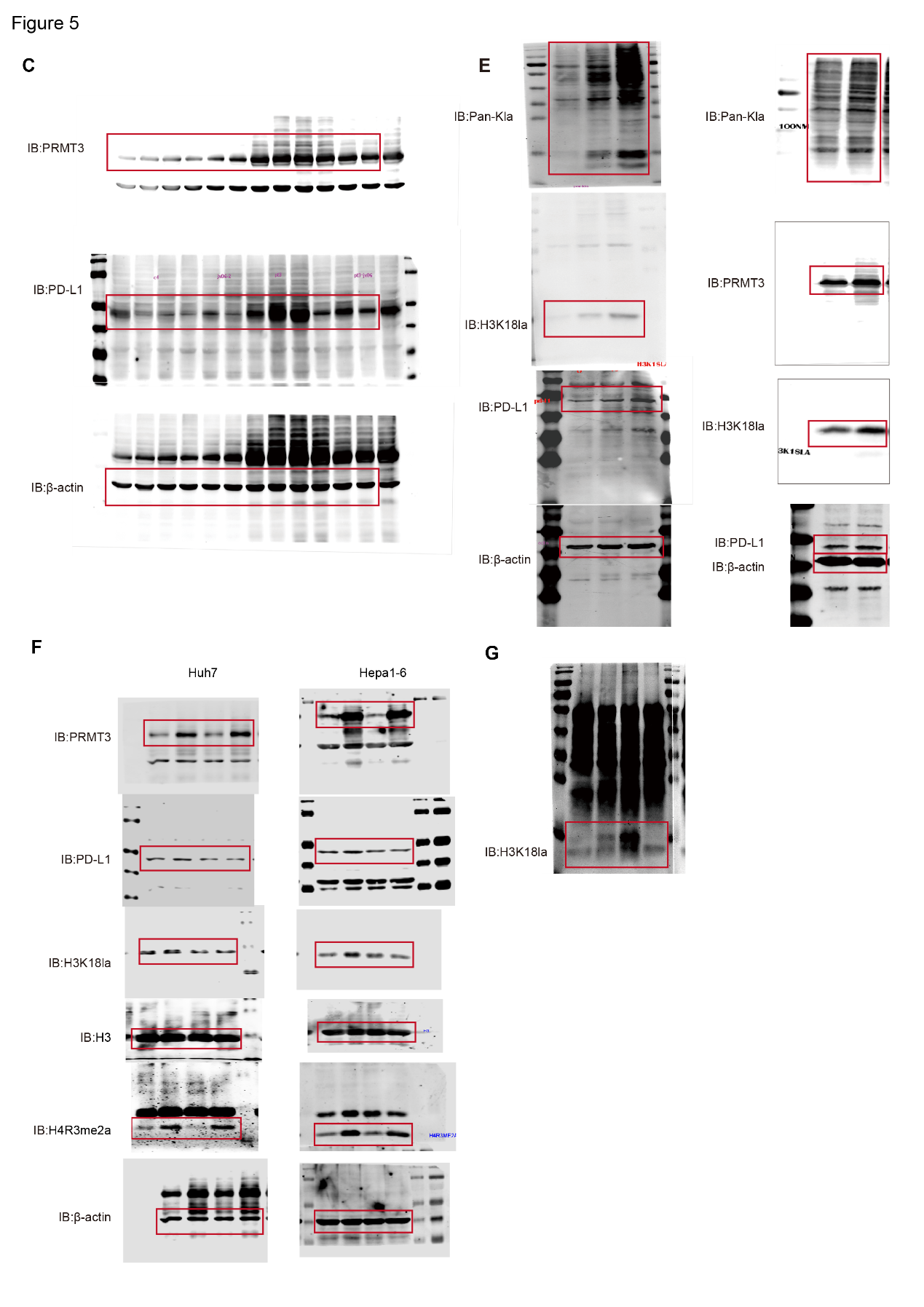


Figure 6


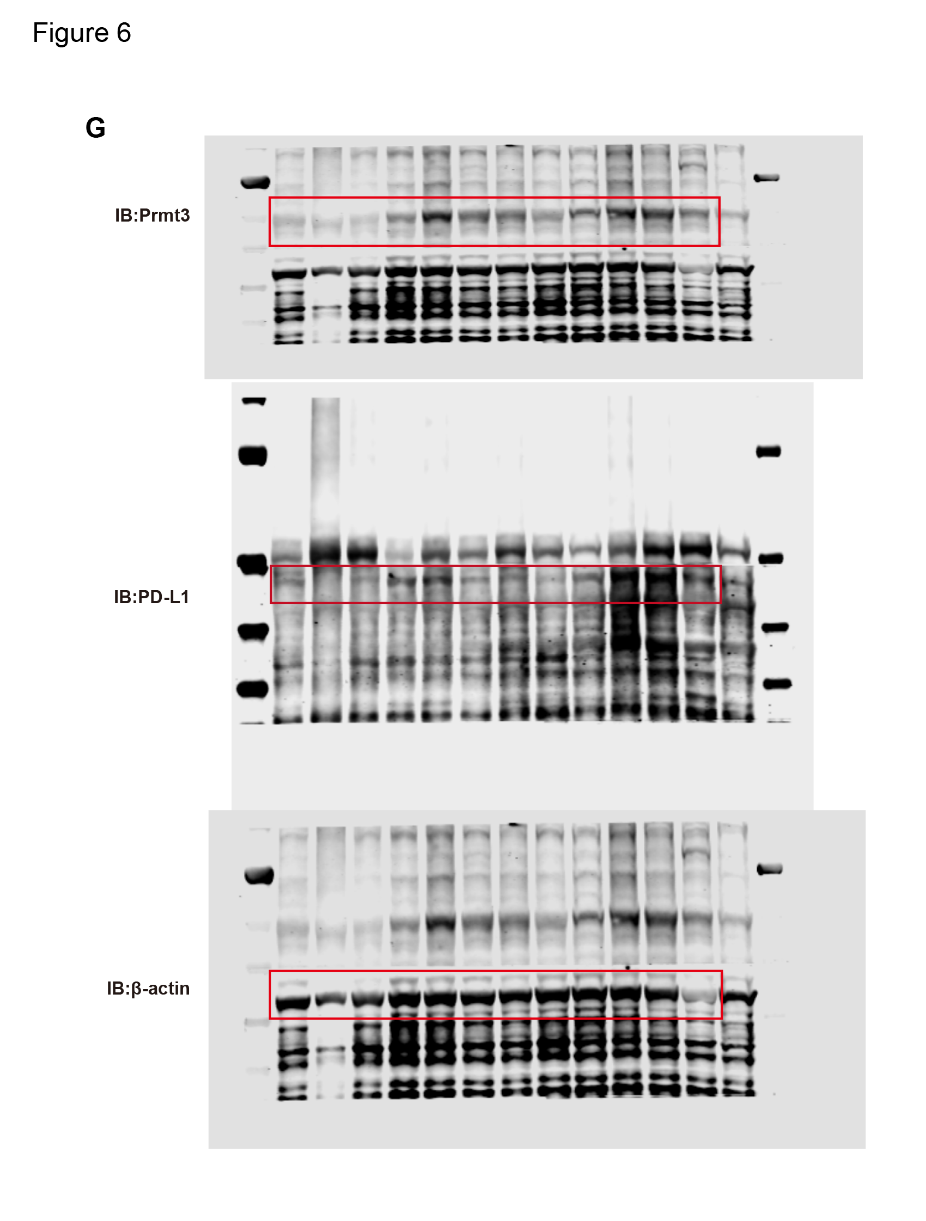


Supplementary figure 2


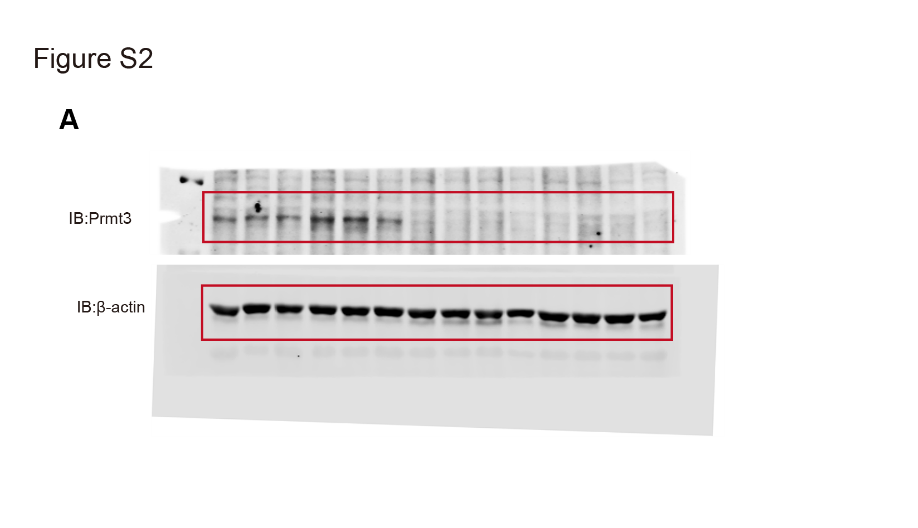


Supplementary figure 3


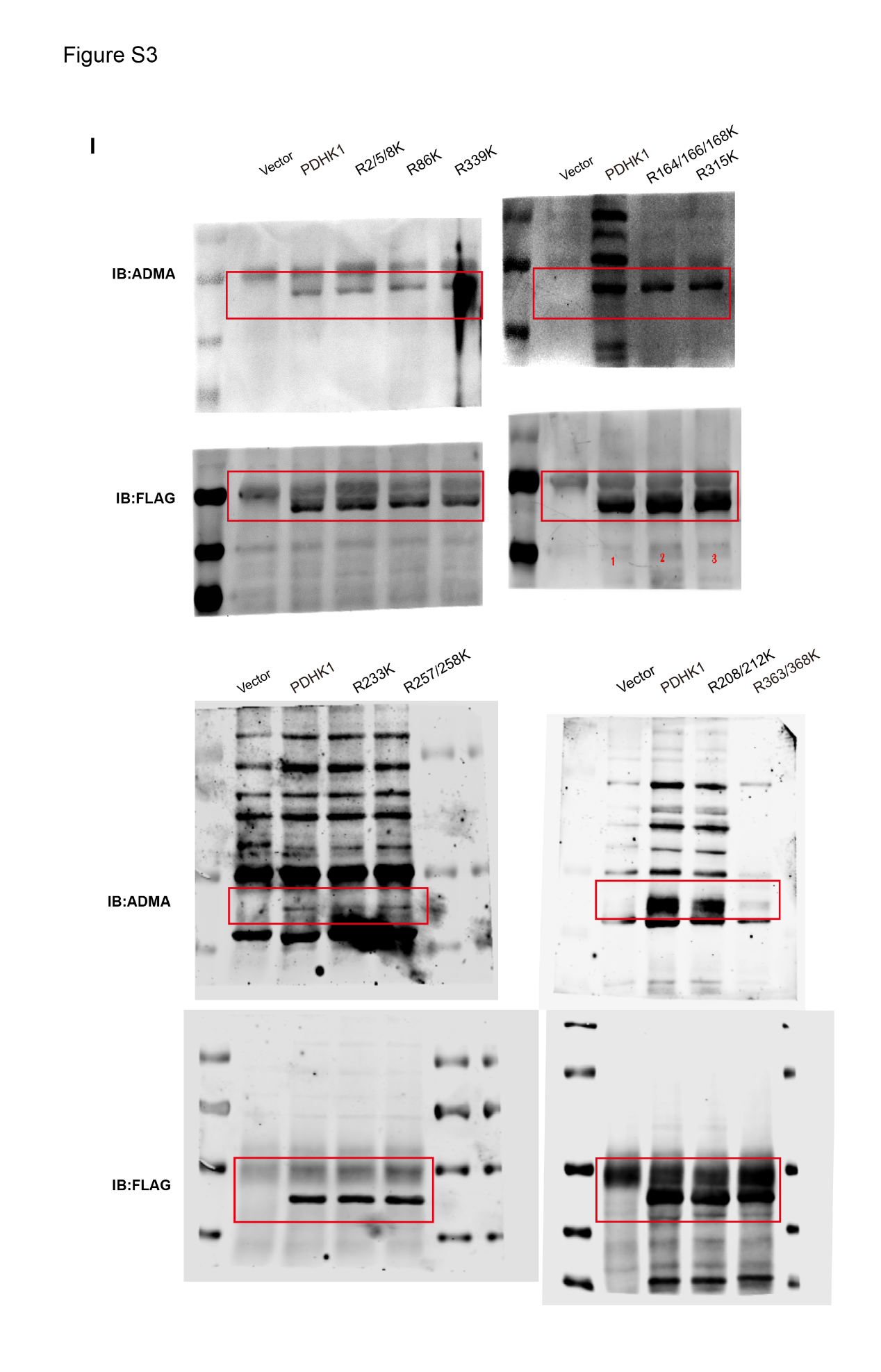


Supplementary figure 4


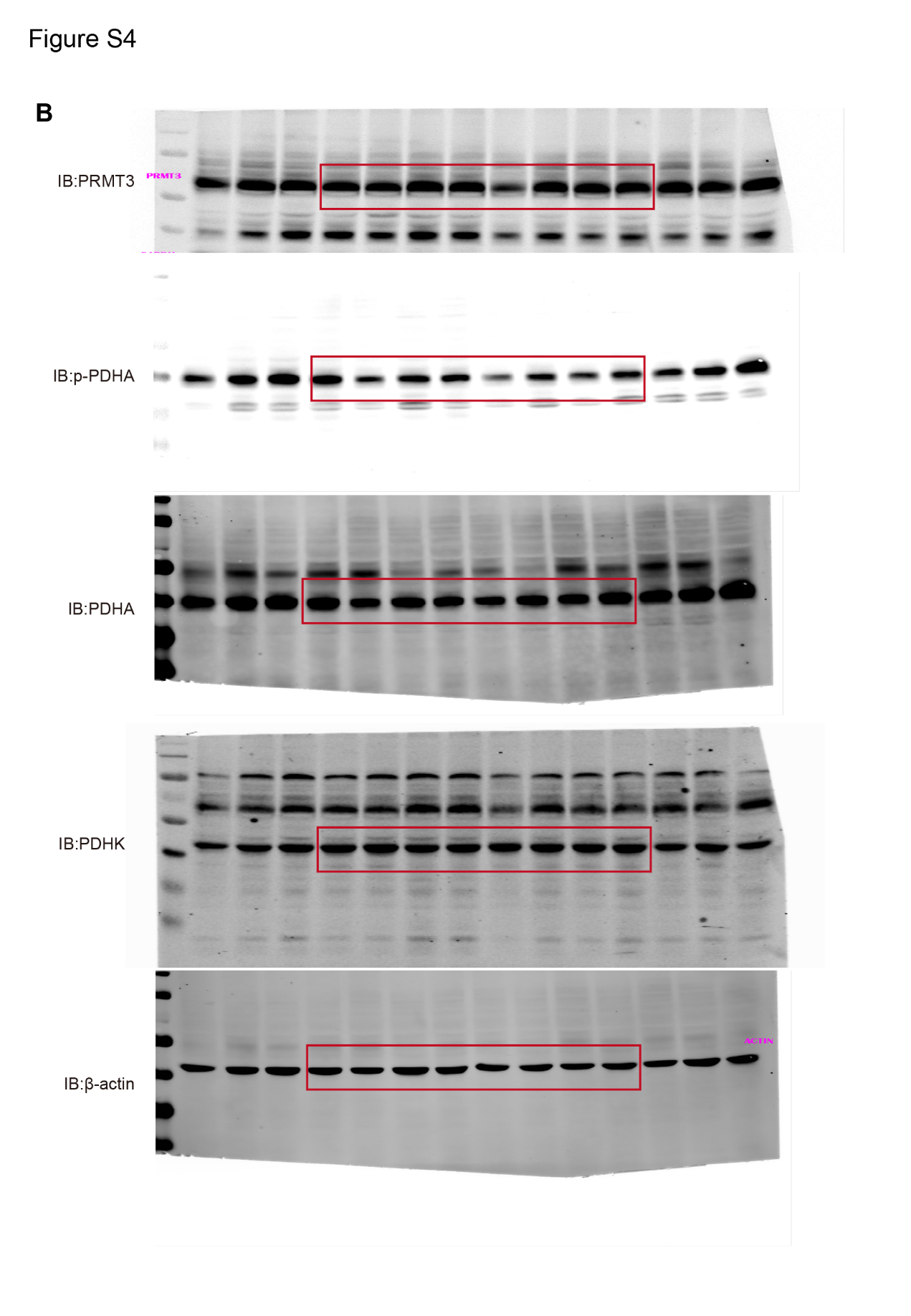


Supplementary figure 5


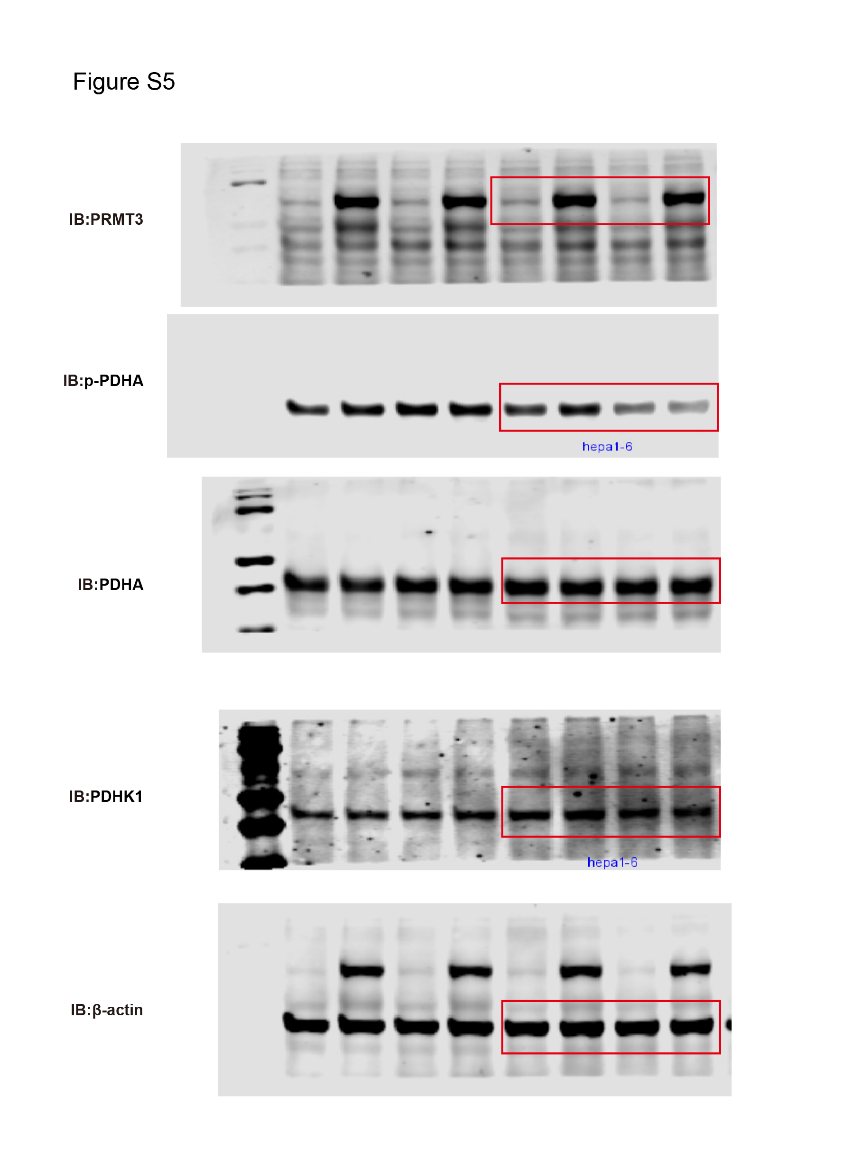

Supplement: Supplementary file 2 — original western blots [file 41419_2025_7482_MOESM2_ESM.docx]
